# Multimodal machine learning for analysing multifactorial causes of disease—The case of childhood overweight and obesity in Mexico

**DOI:** 10.3389/fpubh.2024.1369041

**Published:** 2025-01-07

**Authors:** Rosario Silva Sepulveda, Magnus Boman

**Affiliations:** ^1^Karolinska Institutet, Department of Medicine Solna, Division of Clinical Epidemiology, Stockholm, Sweden; ^2^MedTechLabs, BioClinicum, Karolinska University Hospital, Stockholm, Sweden

**Keywords:** supervised machine learning, unsupervised machine learning, multimodal machine learning, bias, paediatric obesity, obesogenic environment, Mexico

## Abstract

**Background:**

Mexico has one of the highest global incidences of paediatric overweight and obesity. Public health interventions have shown only moderate success, possibly from relying on knowledge extracted using limited types of statistical data analysis methods.

**Purpose:**

To explore if multimodal machine learning can enhance identifying predictive features from obesogenic environments and investigating complex disease or social patterns, using the Mexican National Health and Nutrition Survey.

**Methods:**

We grouped features into five data modalities corresponding to paediatric population exogenous factors, in two multimodal machine learning pipelines, against a unimodal early fusion baseline. The supervised pipeline employed four methods: Linear classifier with Elastic Net regularisation, k-Nearest Neighbour, Decision Tree, and Random Forest. The unsupervised pipeline used traditional methods with k-Means and hierarchical clustering, with the optimal number of clusters calculated to be *k* = 2.

**Results:**

The decision tree classifier in the supervised early fusion approach produced the best quantitative results. The top five most important features for classifying child or adolescent health were measures of an adult in the household, selected at random: BMI, obesity diagnosis, being single, seeking care at private healthcare, and having paid TV in the home. Unsupervised learning approaches varied in the optimal number of clusters but agreed on the importance of home environment features when analysing inter-cluster patterns. Main findings from this study differed from previous studies using only traditional statistical methods on the same database. Notably, the BMI of a randomised adult within the household emerged as the most important feature, rather than maternal BMI, as reported in previous literature where unwanted cultural bias went undetected.

**Conclusion:**

Our general conclusion is that multimodal machine learning is a promising approach for comprehensively analysing obesogenic environments. The modalities allowed for a multimodal approach designed to critically analyse data signal strength and reveal sources of unwanted bias. In particular, it may aid in developing more effective public health policies to address the ongoing paediatric obesity epidemic in Mexico.

## 1 Introduction

Paediatric obesity is a systemic, chronic, inflammatory, and recurrent disease characterised by an abnormal or excessive accumulation of body fat in children up until 19 years (1). At least 50% of children and adolescents affected by obesity continue being affected into adulthood (2), compromising their future health, quality of life, and educational or professional performance (3–6). Paediatric obesity prevalence has more than quadrupled globally from 1975 (4%) to 2016 (18%), regardless of strategies and efforts to control it (7). Paediatric obesity's increasing trend has been found in countries irrespective to their gross income classification (8, 9), with low to middle income countries particularly vulnerable due to the economic burden of obesity which represents on average 8.4% of the annual health spending even for wealthy Organisation for Economic Co-Operation and Development (OECD) countries (5). Mexico is an emergent economy with one of the highest incidences of paediatric overweight and obesity in the world (10). Thus, Mexico's case will be the geographic and demographic focus of this study, using the Mexican National Survey of Health and Nutrition (*ENSANUT: Encuesta Nacional de Salud y Nutrición*) as data source.

**Figure 1:**
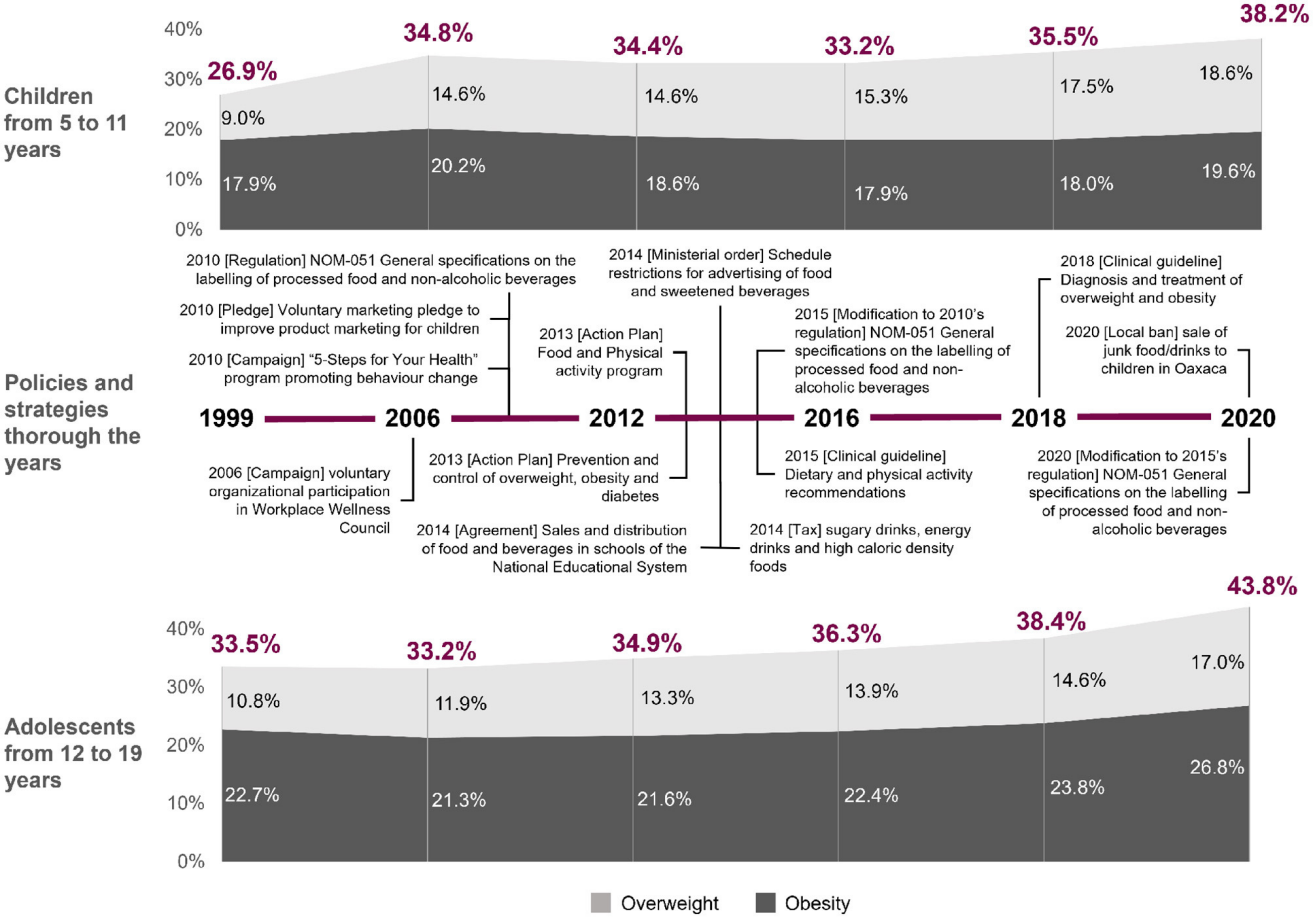
Trends of overweight and obesity in children and adolescents from 1999 to 2020 in Mexico and the policies and strategies implemented over the years. Personal elaboration of earlier reported material (11, 20, 44).

ENSANUT is a probabilistic national survey that works as a regular strategy to assess the status on health and nutrition of the Mexican population and as basis for Public Health policy-making (11). Public health policies and strategies designed from ENSANUT can be observed in Figure 1, and include establishing guidelines for sale and distribution of foods and beverages in elementary schools (2010); restrictions on the television advertisement of high caloric density foods and sweetened beverages for children audiences (2014); adding a 10% tax to junk food to reduce consumption (2014); regulations on front package labelling (2015); updating clinical guidelines for improving timely diagnosis and treatment (2018); and other diverse campaigns or action plans to promote behaviour change (11, 12). Since 1999, the number of children and adolescents in Mexico suffering from overweight or obesity increased by at least 10%, representing 38.2% of children and 43.8% of adolescents by 2020, which could indicate that the implementation of the policies and strategies designed from ENSANUT have failed to control the epidemic (8, 9). Since, to our knowledge, the findings from which these public health strategies and policies were based (11) involved only traditional mathematical and statistical analyses, it is plausible that other methods such as those of machine learning (ML) could provide additional knowledge on the predictors of paediatric overweight and obesity in Mexico, thanks to its capacity to analyse complex and non-linear patterns (13).

Efforts to understand more comprehensively the aetiology of the disease using ML go back to 1995 (14), and a systematic review on ML models to predict childhood and adolescent obesity conducted in 2020 reported that when comparing to the results reached through statistical methods, ML models gave better prediction performance in internal and external validations (15). Studies like the following three suggest that building prediction model using ML, with environmental and phenotypical factors taken into account, is fruitful. Rehkopt et al. (16) used diet and physical activity alongside parental risk factors on the psychological, social, and health aspects. Wiechman et al. (17) made use of demographics, caregiver feeding style and practices, home environment characteristics, and familial characteristics such as social and spousal support. Kim et al. (18) fitted features coming from behavioural patterns of teenagers like family wealth, smartphone use, amount of pocket money, academic performance, and quality of sleep.

An obesogenic environment is characterised by surroundings, opportunities, or conditions of life, external to the individual, which promote overweight or obesity (19). The paediatric population's environment is almost completely regulated by the adults who are part of their life and their dwelling (10). Hence, exploring exogenous factors and studying them as a whole is key to understanding the aetiology of paediatric obesity more comprehensively (10, 19, 20). These type of studies could also promote creating public health strategies to address obesogenic environmental factors, which are currently rare (11, 19, 20). Out of the different ML methods, multimodal ML models can be particularly useful for studying exogenous factors of paediatric obesity as it lets us process and fuse information from multiple data modalities. Modalities can convey different sources of data: applications in health care frequently use this approach to predict a specific health outcome (21). A supervised multimodal ML pipeline includes early and late fusion approaches (22). Early, or feature, fusion involves concatenating the data from the different modalities after the pre-processing stage and before training the model. Late, or decision, fusion involves selecting the features, training and validating the models individually, and then using a series of techniques to combine the predictions of each modality's best performing methods (23).

Our aim is to explore if multimodal ML can enhance, against a unimodal baseline (see Section 2.6.2), identifying predictive features from obesogenic environments and investigating complex disease or social patterns using the latest ENSANUT available to us (ENSANUT-18) as data source. The study includes two multimodal ML pipelines with supervised and unsupervised approaches, respectively (24). Our approach could help identify clusters that may not be targeted yet by public health initiatives or could aid identifying the most predictive features from the household that promote the development of the disease. Furthermore, the knowledge extracted could be used to design early interventions in the form of clinical guidelines (15). This makes it possible to identify the most predictive features from the child or adolescent's environment and to investigate comprehensively explanatory patterns for the development of paediatric overweight and obesity in Mexico.

## 2 Materials and methods

### 2.1 Study outline

**Figure 2:**
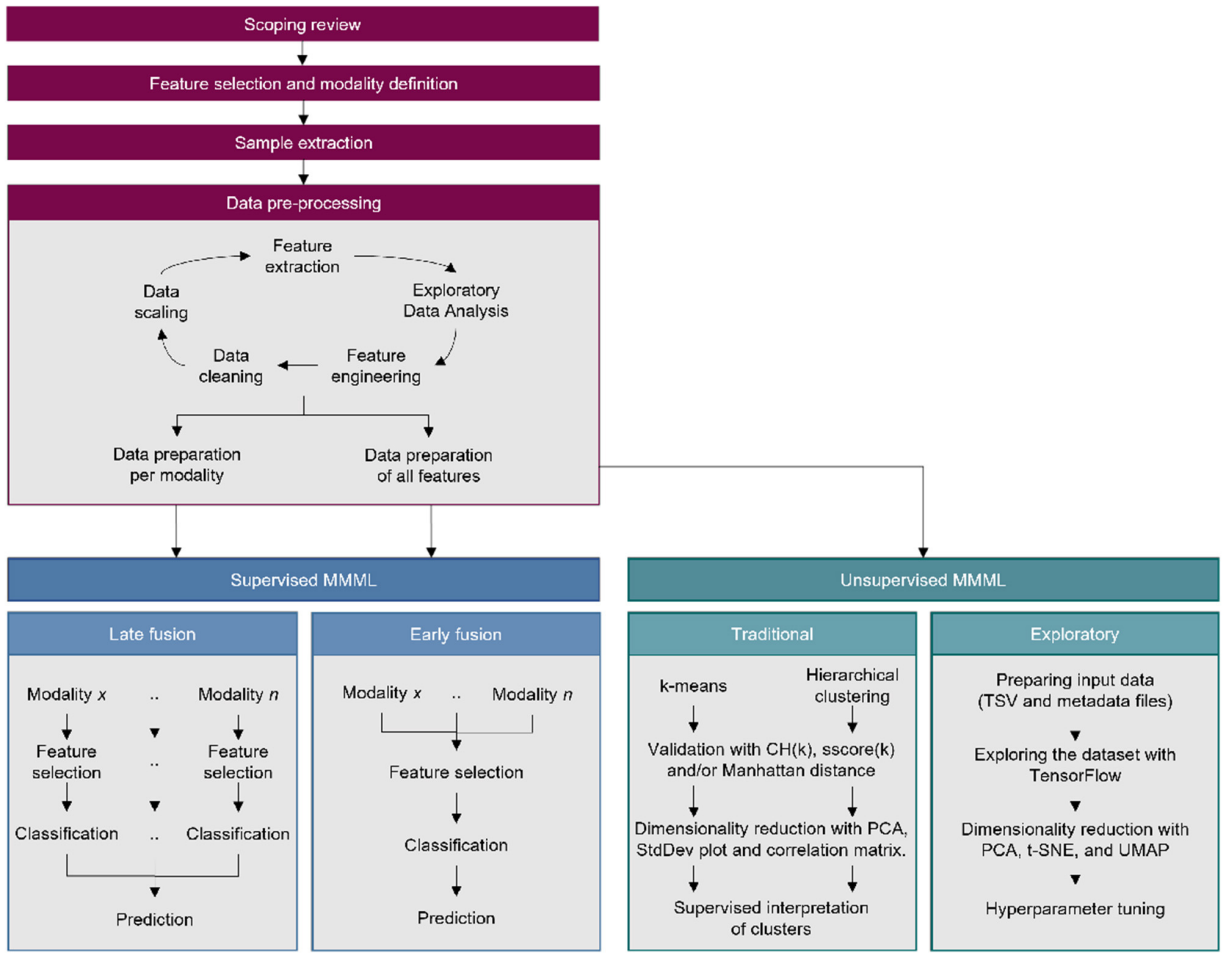
Complete study outline which includes a data preparation phase preceding the supervised and unsupervised machine learning pipelines.

As shown in Figure 2, the study consisted of three phases: a preparation phase and two separate multimodal ML training phases (also called *pipelines*). The preparation phase had the goal of producing the datasets to train the models in the consequent phases. The multimodal ML pipelines adhered to Carbonell et al. (24) for its comprehensiveness and scalability (25), and for its critical bias detection features. Additionally, for replication purposes, we have made available a GitHub repository containing all scripts used to prepare the datasets and run the ML pipelines: https://github.com/rosarioss/multimodal-paediatric-obesity.

### 2.2 Scoping review

The first step was to perform a scoping review to facilitate an informed selection of the features to include in the study. The review focused on identifying studies where the ENSANUT database was used to explore feature correlations on topic. Journal articles that used ENSANUT data as their primary source, published in English or Spanish, at any time, were included. Six databases were used: Medline, Web of Science (WoS), Cumulative Index to Nursing and Allied Health Literature (CINAHL), Scientific Electronic Library Online (SciELO), Global Index Medicus from the World Health Organisation (GIM/WHO), and Virtual Health Library from Pan American Health Organisation (VHL/PAHO). The databases were selected for their extensive variety of health informatics-related topics, or because of their inclusion of Mexican or Latin American journals (SciELO and VHL/PAHO). Detailed criteria can be found in the Supplement, Section 1, including a PRISMA chart in Supplementary Figure S1.

### 2.3 Feature selection and modality definition

**Figure 3:**
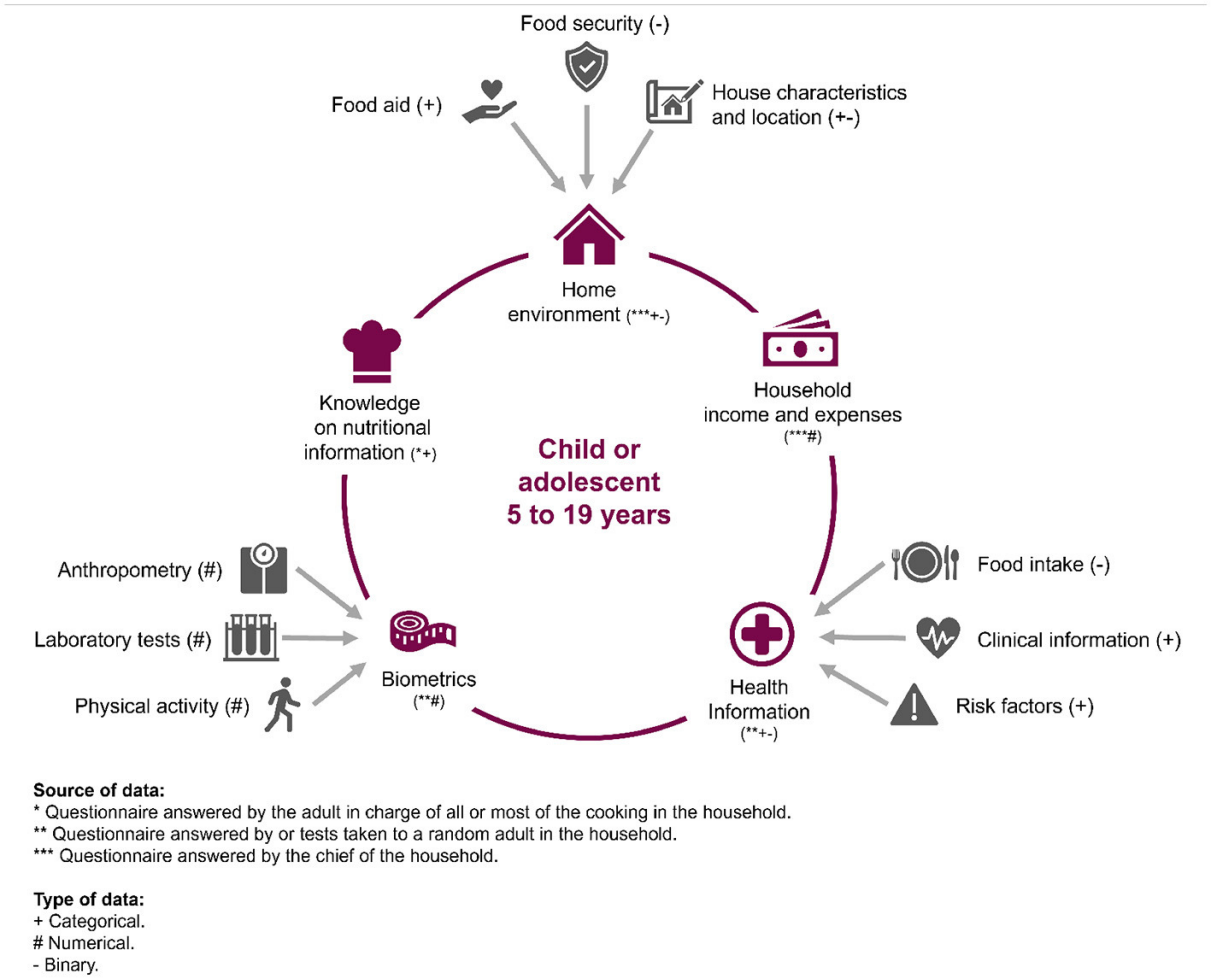
The five modalities defined for the study, with their corresponding sources and types.

Following the scoping review, the features in each ENSANUT questionnaire were examined to identify those that should be “Definitely included” (271 of them), “Definitely excluded,” and “Neutral.” Since the focus of the study was analysing obesogenic environments, questionnaires that focused directly on the child or adolescent were excluded from the feature selection process. The questionnaires included referred either to a person that could have a direct influence on daily habits or on the home environment *per se*. This could either be represented by the adult who usually oversee the preparation of food or an adult randomly selected from the household by the ENSANUT-18 interviewers. The “Definitely included” features were organised in five modalities: home environment, household expenses and income, health information, biometrics, and knowledge on nutritional information (summarised in Figure 3 and detailed in Supplementary Figure S3). Each modality is further explained in Section 2.5 to avoid repetition. Criteria for categorising the features summarises as follows:

- Definitely excluded:

° features unrelated to the environment of the child or adolescent,
° features that focused directly on the child or adolescent,
° multicollinear features,
° features containing more than 35% NaN values;

- Definitely included:

° features identified in the scoping review,
° Other environmental features;

- Neutral:

° Redundant features in view of another “Definitely included” feature,
° Remainder features with no natural inclusion into the other two categories.

### 2.4 Sample extraction

The sample population dataset for this study was built from an anthropometric data questionnaire applied to children and adolescents. The study population includes female or male individuals from 5 to 19 years of age (60–228 months) at the time of ENSANUT-18 data collection. The first step in the data selection process was to exclude pregnant individuals, due to potentially altered BMI, and those who were missing anthropometric data. Afterwards, metadata required to identify the study population (such as multi-level unique keys) and features to calculate BMI-for-age (Weight, height, age in months, gender) were extracted and prepared into the sample population dataset. The International BMI-for-age curve formula (26), defined by the World Health Organisation (WHO) to diagnose paediatric overweight and obesity (27), was applied to calculate the BMI-for-age and interpret it. WHO's BMI-for-age measures for children and adolescents from 5 to 19 years consist of sex-specific age curves computed by percentiles or *z*-scores. The interpretation of the *z*-score cut-offs, based on the number of standard deviations from zero, were used as labels for the model training (28–30):

- Underweight: less or equal to –1SD,
- Normal weight: more than –1SD and less than +1SD,
- Overweight: more or equal to +1SD and less than +2SD,
- Obesity: more or equal to +2SD.

After the initial data cleaning of the sample population dataset, a total *n*= 10,301 observations remained. However, the actual dataset size to train each of the models depended on the modal approach. For the late fusion pipeline, each modality had different sample and dimension sizes. For the early fusion pipeline and the unsupervised pipelines, three dataset with equal sample size for different dimension size were required: a numerical dataset, a categorical dataset, and one containing both. The first two were used to process separately when using Mean Squared Error as a dimensionality reduction technique during the unsupervised traditional approach. The latter was used for the supervised early fusion and for both unsupervised approaches. See next section for details on the final dataset size for each modality.

### 2.5 Data pre-processing

Data pre-processing included a cyclical process of feature extraction, exploratory data analysis, feature engineering, data cleaning, and data scaling until the dataset was considered ready for data preparation. Feature extraction consisted of importing the “Definitely included” features from the corresponding questionnaire and concatenating them to form one dataset for each modality. The dataset then underwent a general exploratory data analysis that looked for general data patterns such as distribution of each feature and identifying NaN values, with the purpose of identifying Feature engineering requirements. Feature engineering involved different strategies, such as computation of numerical values or creation of dummy variables for categorical features, depending of the modality (see Sections 2.5.1 through 2.5.5 for details). Data cleaning included an additional step to identify features that should be “Definitely excluded” based on insufficient data quality. This was defined as having a Pearson correlation of ≥0.70 (multicollinearity) or containing more than 35% NaN values. If a feature was discovered to have a multicollinear relationship with another feature, one of the features were dropped. The criteria for keeping features considered the amount of information one questionnaire item provided as compared to the other one, see Sections 2.5.1 through 2.5.5 for details. Ambiguous answers such as “I do not know” or “I do not remember” were processed as NaN values as well. The dropping criteria for NaN values followed these rules: if the feature had more than 35% NaN values, the whole feature (column) was dropped. If it had < 35% NaN values, then only the rows that had NaN values were dropped. The exception to dropping rows was if the dataset forced *n* below the acceptable level given by the Cochran equation for infinite values (31):


(1) $$\begin{array}{l} n=\frac{z^{2}\times \rho \left(1-\rho \right)}{\epsilon^{n}} \end{array}$$

where: *z* = *z*-score, ϵ = margin of error, *n* = population size, and ρ = population proportion.

For the Mexican population of ~32.5 million 5–19 year olds, with a margin of error of 2%, a confidence interval of 95% and an overweight- or obesity-affected population proportion of 42.3%, the minimum acceptable size of each dataset was *n* = 2,344.

Data preparation consisted once more on dropping every observation that had at least one NaN value (imputation was avoided to prevent generating bias), assigning each observation a random group number from 1 to 5 in preparation for using Leave-one-group-out (LOGO) Cross-validation (CV), and performing feature scaling (for numerical features) or creating dummy features (for categorical features). The randomised groups are needed for LOGO-CV so that one of the groups can be excluded from the training pipeline and the model can be tested against and independent and unseen fold. This helps prevent overfitting and inflated performance estimates, even when there's correlation or dependency within the groups. In contrast, *k*-fold cross-validation randomly splits the dataset into *k* parts without regard for any inherent group structure, which may lead to data leakage when groups are present, since multiple records from the same subject may end up in both the training and test sets.

#### 2.5.1 Modality 1: home environment

This modality included features about the environment where the child or adolescent was growing up. It comprised sociodemographic data, data about subscription to food aid programs, food safety situation (results of ELCSA), physical characteristics of the house (e.g., conditions in which food is prepared), as well as location (locality size and type). Feature engineering involved three main transformations, the most significant of which was the calculation and interpretation of the ELCSA into food safety, as well as mild, moderate, and severe insecurity in the home. Two features (“fuel type used to cook” and “stove type”) were dropped due to multicollinearity with another feature (“has gas stove”); only the latter feature was kept. Since no dimensions had more than 35% missing values, only rows with NaN values were removed. Label distribution remained similar to the sample dataset, with suffering from overweight or obesity at 42.5%, as well as the representation of the sample demographic metadata after groups were created for LOGO-CV. After pre-processing, the data set included 39 dimensions and 9585 observations.

#### 2.5.2 Modality 2: household expenses and income

This modality included features about the monthly income of the household and food or health expenses per category of product or service consumed. Feature engineering involved three main transformations, the most significant of which was the calculation of the proportion that a specific product or service represented of the total household expenses, measured in either food or health expense. Other aspects were analogous to those of Modality 1. Because Modality 2 contained only numerical features and the distribution of the label in its SD format was non-Gaussian, the feature scaling strategy involved standardising the features. After pre-processing, 21 dimensions and 7, 819 observations remained.

#### 2.5.3 Modality 3: health information

‘ This modality included features about current health status, personal and familial health history, health risk factors (Table 1), and food intake patterns of a random adult living in the same household as the child or adolescent. The demographic features of the adults selected at random included showed that 62.3% were between 30 and 49 years old, predominantly female (57.6%), and predominantly the parent of the child or adolescent (68.7%). Demographic details are provided in Supplementary Figure S7, whereas feature engineering is detailed in Supplementary Figures S12, S13. The latter involved calculation of the kinship. Worth noting is that since the relationship registered in the database was that of a resident of the household towards the “household chief,” a series of functions needed to be created to translate that into kinship towards the study population. Due to this situation, certain limitations when computing the kinship were identified, for instance if the adult selected at random was the son or daughter of the chief and the child or adolescent was the grandchild, it was not possible to discern if the adult was the parent or uncle/aunt. Nevertheless, kinships obtained provided interesting information presented in the Section 4.

**Table 1:** Documented risk factors for paediatric overweight and obesity, per category.

**Category**	**Risk factors**
**Dietary patterns**	**Fast food consumption (4, 32, 52), sugary beverages consumption (2, 53), snack foods consumption, portion size, and availability and accessibility of unhealthy food (4, 32, 33, 52, 54)**
Physical activity	Level of physical activity, sedentary behaviour, basal metabolic rate, time in front of screens (1, 4)
Genetics	Genetic predisposition (4), genetic conditions such as hyperphagia (55)
Family dynamics	Parenting style, parental physical exercise habits, parental feeding style, family mealtime structure (4, 34, 56), grandparent co-residence in the household (57–59)
Environment	School policies (4, 60), parents' work-related demands (42, 56), fast-food industry (2, 61), regulation of marketing strategies for unhealthy foods (4), safety of walking/cycling routes (4), store density (54)
Socio-cultural influence	Food-as-reward related beliefs (4), perception of being a normal stage of growth (1)

The feature containing the number of working hours per week, which was originally a numeric feature, was converted to binary for two reasons. First, to evaluate if the person was working more or less than the law-regulated maximum of 40 h per week. Second, because through the experimental data analysis it was discovered that most answers were either 40 or 50, giving the data a semi-categorical aspect from the start. Twelve features regarding the question “Which healthcare institution are you affiliated to?” were dropped due to multicollinearity with their correspondent items on the question “Where do you go to receive healthcare?” (see list of features dropped in Supplementary Figures S12, S13). The latter features were kept since, by default, included the information on the healthcare institution affiliation. After pre-processing, the data set included 113 dimensions and 3, 383 observations.

#### 2.5.4 Modality 4: biometrics

This modality included the numerical features about the anthropometric measurements and laboratory results of the same randomly selected adult as in Modality 3. Feature engineering involved six main transformations (see Supplementary Figures S12, S13). The feature regarding “Total cholesterol level” was dropped due to multicollinearity with “HDL cholesterol” and “LDL cholesterol” levels. The feature on “Total cholesterol” was dropped since the type of cholesterol provided more information than the total cholesterol. Pre-processing produced 19 dimensions and 4, 419 observations.

#### 2.5.5 Modality 5: knowledge on nutritional information

This modality included the results of a test about the nutritional information on the packaging. The responding adult was the one that oversaw most of the cooking in the household. The test included questions about usage of different types of nutritional information, labels or legends printed in commercial products, as well as questions on the supposed importance of different nutrients such as carbohydrates, proteins, salt, fats, trans-fats, total energy, etc. About the food intake patterns, previous studies had found that a pattern of healthy eating could not be defined based on the numerical self-reported portions in the survey (32–34). Reasons for this could be under- or over-reporting of food intake based on social pressure (8, 34), or simply the challenge of answering the survey, as it required to remember the portion sizes based in grammes or millilitres per food group (e.g., fruits) and food item (e.g., bananas) eaten during the past 7 days (8). Hence, to avoid possible sources of noise or bias, only the binary version of those questions, based on WHO's daily food intake recommendations, were included.

The demographic features of the adults in this modality shows that 65.1% were between 30 and 49 years old, predominantly female (58.7%), and predominantly the parent (70.5%). The largest challenge in the feature engineering was that questions about the correct identification of three products had to be dropped, due to two thirds of the values missing. The feature regarding the question “Do you use the nutritional information of a product to compare their nutritional value?” had to be dropped due to multicollinearity with the question “Does the nutritional information of a product affect your buying decision?”. The latter was kept since our main interest was to know if the product reached the home environment of the children or adolescent. After the pre-processing, the dataset included 70 dimensions and 4, 399 observations.

### 2.6 Supervised methods

The supervised multimodal ML pipelines used in this study included early and late fusion approaches (Figure 2); Sections 2.6.1, 2.6.2 detail these, respectively. The pipelines display some differences to the classical approach to multimodal ML. Firstly, in the latter, each data modality corresponds to different sources that capture the same reality (e.g., video and audio from the same recording). The modalities defined in this study instead correspond to different environmental sources that could affect the study population's behaviour. Second, the classical approach typically uses data from a micro-level of social analysis (individual identity, motives, and cognition) to predict an individual's label in the model. Since the present study aims to study obesogenic environments, the data used corresponds to a meso-level of social analysis (organisations and groups, such as family members in the same dwelling) to predict an individual's label (35).

Both early and late fusion pipelines used the same four ML methods; which are supported by a review on ML for obesogenic environments among others (15). Decision Trees and Random Forest methods can both handle continuous features that have been scaled and categorical data encoded as binary features. The k-Nearest Neighbour (kNN) was appropriate because as a non-parametric algorithm it makes no assumptions on the distribution of the data. Finally, a Linear Classifier with Elastic Net Regularisation (Elastic Net) offered the calculation of a Logistic Regression Coefficient that could also be used to evaluate feature importance and its positive or negative predictive association (36). Logistic Regression coefficients were used to analyse predictive patterns of the features and compare their predictive importance when using early fusion (unimodal) and late fusion (multimodal) approaches.

For analysing the performance of the supervised models, several metrics were employed, including Area Under the Receiver Operating Characteristic (AUROC), Recall, and greater-than-chance analysis. AUROC was used to compare performance between classifiers and between the training and validation sets of the same model and represents the probability that if given a randomly chosen positive and negative sample, the model will rank the positive higher (36). Recall is one of the four common performance measurements calculated from a confusion matrix (37), measuring the ability to identify all the true positive predictions. Greater-than-chance analysis was used to compare the Recall value, representing the random probability of being a True Positive as 0.25.

The classifier hyperparameters were tuned using Exhaustive Grid Search (GridSearchCV) method from Scikit learn. This method tries every possible combination for a set of given hyperparameters, hence it is both comprehensive and effective in finding near-optimal hyperparameters. Details and the search grids can be found in the Section 5 in Supplementary methods and Supplementary Figures S8, S11. All methods were tested using a Jupyter Notebook running Python 3.9 and the Pandas, NumPy, scikit-learn, Pickle, Seaborn and Matplotlib libraries, and the code is publicly available. In particular, NumPy was used to run mathematical, statistical, logical, and random simulation operations that required using a multidimensional array object, for randomly selecting households and members of those households. Since the ENSANUT-18 database is openly available, this allows for reproduction and also for later databases to be tested in the future.

#### 2.6.1 Multimodal data experiments

Multimodal data experiments exclusively involved the Supervised Late Fusion pipeline. Late fusion, or decision-level fusion, involves selecting the features, training, and validating the models individually and then using a series of techniques to combine the predictions of each modality's best performing classifiers. One of the benefits of late fusion is that it allows for having different size of observations and data types in each modality, while early fusion require the same number of observations before concatenating the data and building the models. The techniques, or rules, used to combine the predictions were the following:

- Maximum rule: get max value between classifiers,
- Sum rule: sum the scores from each modality and calculate the average,
- Product rule: get product value between classifiers,
- Weight criterion: get ratio of each modality and compute the weighted scores,
- Rule-based: choose classifier according to validation confusion matrix.

#### 2.6.2 Unimodal data experiments

Unimodal data experiments involved both the Supervised Early Fusion pipeline and the Unsupervised pipelines. Early fusion, or data-level fusion, involves concatenating the data from the different modalities after the pre-processing stage and before training the model. In terms of training and validation, this can be considered a unimodal strategy since all the features are used together to train the model, as a single modality. In this case, all the features from Modality 1 through Modality 5 were included and feature engineering involved all of the transformations described above, in Sections 2.5.1, 2.5.2, 2.5.3, 2.5.4, 2.5.5.

The features were first concatenated and then processed together in terms of dropping columns or rows of missing values. The biggest difference was that the threshold for dropping columns with missing values had to be lowered to 30% because the representativeness of the size of *n* was compromised (see Section 2.5). Label distribution remained similar to the sample dataset, as well as the representation of the sample demographic metadata after random groups were created for LOGO-CV. Three different manipulations were required after this point, creating three separate datasets: one with only numerical features, another one with only categorical features, and the last one containing both. The first two were used to process separately when using Mean Squared Error as a dimensionality reduction technique during the unsupervised classical approach. The last was used for the supervised early fusion and for both unsupervised approaches. For the categorical features, dummy features were created, while the numerical features were normalised. After the pre-processing, the three datasets contained 2, 467 observations and varied in dimension size: the categorical consisted of 183, the numerical of 28, and the multimodal thus of 211 dimensions.

### 2.7 Unsupervised methods

This study's unsupervised ML pipeline includes classical clustering methods and visual examination using TensorFlow Embedding Projector, where the adjustable parameters were manually tuned until interesting patterns were found. We have made everything publicly available so that readers can visualise and interact with the data themselves. In this way, the dimensionality reduction algorithms produce separations in the cluster analyses (38). Traditional clustering methods included k-Means and hierarchical clustering. The former clusters data trying to separate samples in *n* centroids of equal variance, while the latter represents the hierarchy of clusters as a tree with the root containing all the samples and the leaves containing only one sample (36).

Dimensionality reduction was used to adjust the models in terms of pattern recognition. The optimal number of clusters was calculated using two techniques measuring cluster tightness and separation: the Calinski-Harabaz score (CH Index) and the silhouette score. The CH Index has reduced sensibility to monotonicity, varied cluster densities, subclusters, and skewed distributions (39). The silhouette score offers a more robust technique for handling noisy data than CH Index, since it considers intra-cluster distance and different-nearest-cluster distance (24, 40). Dimensionality reduction methods used were Principal Component Analysis (PCA), t-distributed Stochastic Neighbour Embedding (t-SNE), Mean Squared Error (MSE), and Uniform Manifold Approximation and Projection (UMAP). PCA aids in identifying the most meaningful patters among redundant features or noise, while t-SNE improves the visualisation of high-dimensional data. UMAP is a manifold technique that competes with t-SNE in visualisation quality but with a better run-time performance and arguably in mathematical grounding. Finally, MSE measures how close a regression line is to a set of data points: we dropped features that had variability close to zero ( ≤ 0.14), to reduce noise in the data. As stated above, the numerical and categorical datasets were used to process separately when using Mean Squared Error as a dimensionality reduction technique during the unsupervised classical approach.

### 2.8 Ethics approval statement

All information in ENSANUT-18 has been pre-processed to remove sensitive data before made publicly available and does not include any open text answers that may give the identity of the participants away. Special consideration has been taken to limit the impact of unwanted bias, including handling multi-collinearity, avoiding imputation of missing values, and analysing the context in the original Spanish in which the question was phrased and what answers were offered, when selecting the features to include in the models.

## 3 Results

### 3.1 Scoping review

The 21 articles that met the inclusion criteria of the scoping review were analysed in four categories: dietary patterns (38.1%); physical, social, cultural, economic, or political environment (28.6%); epidemiology (23.8%), and family dynamics (9.5%). The article that analysed the highest number of features (45) out of all reviewed was in the first category. All the studies into dietary patterns found were based on the Food Frequency Intake questionnaire used in ENSANUT 2006, 2012, 2016, and 2018. Most studies concluded that the questionnaire overreported healthy foods and underreported unhealthy foods, especially so in the cases where the person answering the questionnaire was suffering from overweight or obesity. In the second category, relevant sociodemographic features included the region where the subject lived, type of locality (rural or urban), size of locality, Socio-Economic Strata (SES), enrolment in governmental food aid programs, and the result from level of Food Security obtained from the Latin American and the Caribbean Food Security Survey (*ELCSA: Encuesta Latinoamericana y del Caribe de Seguridad Alimentaria*). In the third category, descriptive statistics and logistic regression models were the most common analysis strategies. Only one study proposed a stratified approach of interpreting reduced or excess weight. Regrettably, it was more beneficial for identifying the causes of children with an underweight status than for those with excess weight. In the fourth category, we only found studies that analysed the relationship of a social factor with maternal characteristics (professional situation or breastfeeding practices). The full results of our scoping review can be found in structured form in Supplementary Figure S2.

### 3.2 Supervised multimodal pipelines

#### 3.2.1 Early fusion

**Figure 4:**
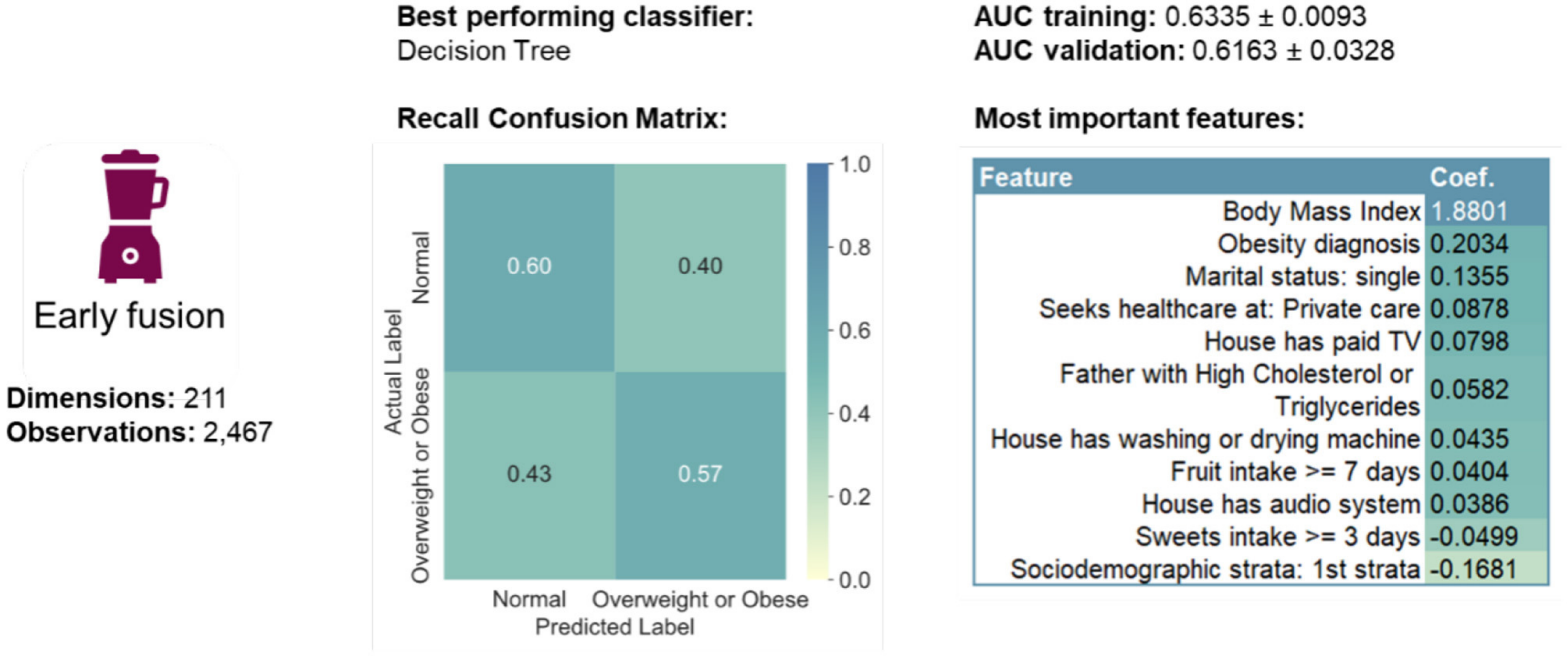
Summary of early fusion performance measures.

The best performing classifier in the early fusion approach was the Decision Tree (Table 2 and Figure 4). Out of the top five highest logistic regression coefficient feature importance scores, four were features of the randomly selected adult in the household (BMI, obesity diagnosis, being single, and seeking private care), while the fifth was related to the house environment (having paid TV). Note that ~85% of the single-person households in Mexico are single-mother ones.

**Table 2:** Performance of the four machine learning methods used for the early fusion approach, during training and testing.

**Classifier**	**Training AUROC**	**Testing AUROC**
Elastic Net	0.6429 ± 0.0070	0.6021 ± 0.0293
k-NN	0.5662 ± 0.0048	0.5575 ± 0.0065
**Decision Tree**	**0.6335** ±**0.0093**	**0.6163** ±**0.0328**
Random Forest	0.9750 ± 0.0012	0.6088 ± 0.0234

The best quantitative score was obtained by the Decision Tree method, in bold. Random Forest was overfitting heavily.

#### 3.2.2 Late fusion

**Figure 5:**
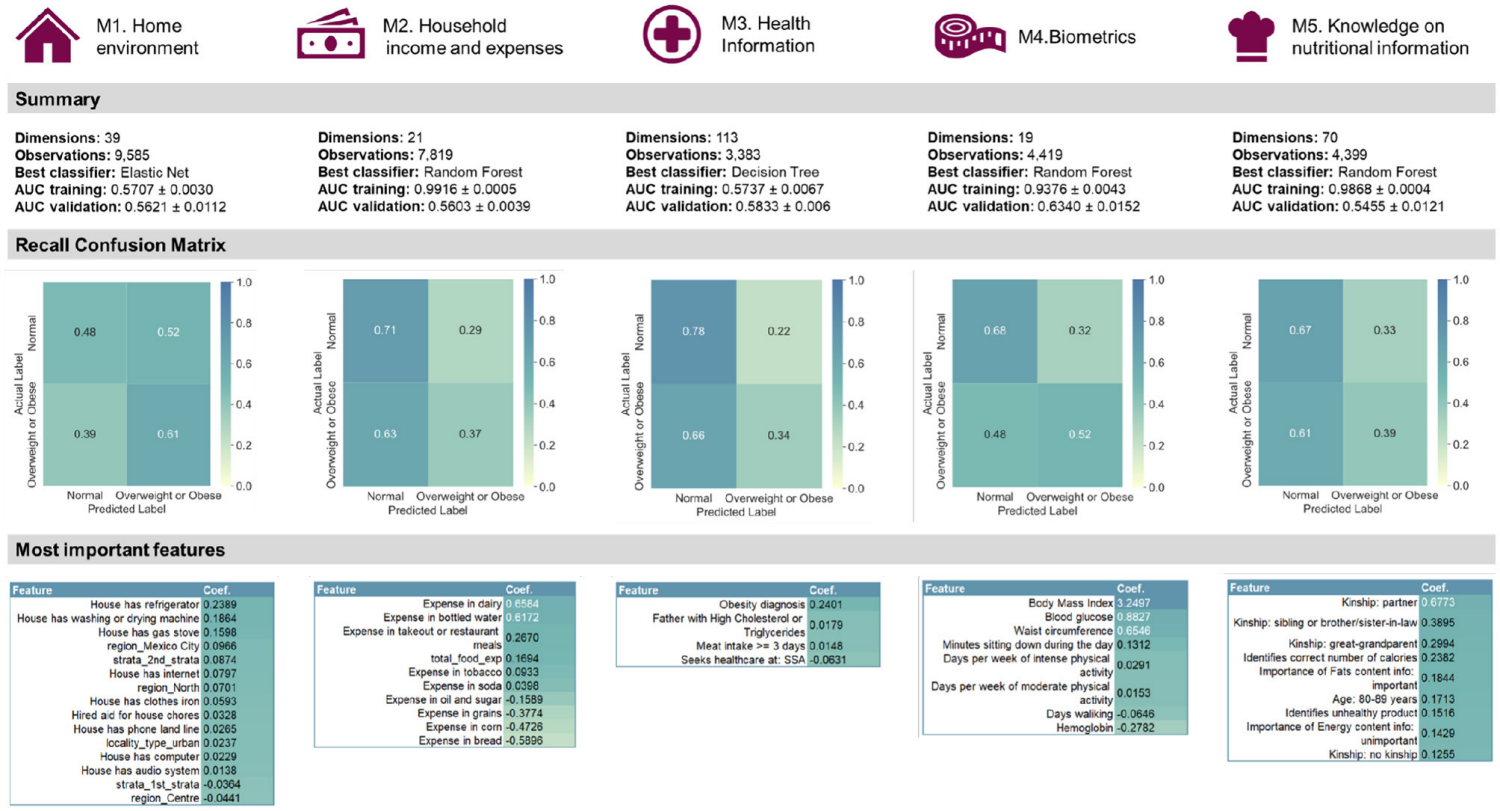
Summary of the quantitative late fusion approach and its feature importance results.

**Figure 6:**
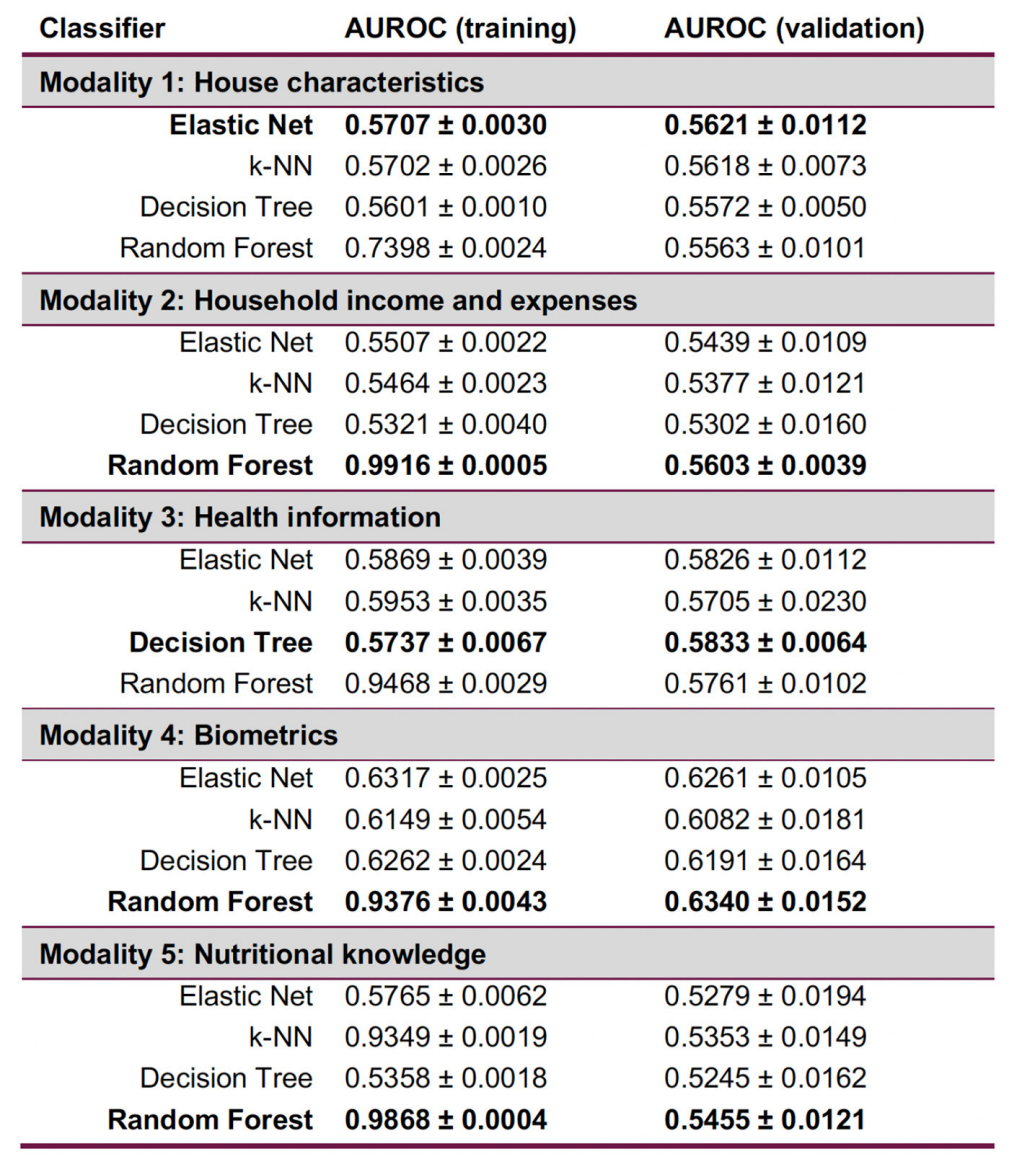
Late fusion results per modality, with each best-performing validation ML method in bold. Random Forest is consistently overfitting. Expressions of worst-case time complexity are detailed in the Supplementary Section 4; see Supplementary Figure S9.

The best performing classifier (Figure 5) varied per modality (Figure 6): Elastic Net for Modality 1, Decision Tree for Modality 3 and Random Forest for Modality 2, 4, and 5. In terms of the recall confusion matrix, Modality 1 had the highest performing predictor for the Overweight or Obese label (0.61), while Modality 3 had the highest value for the Normal label (0.78). Compared to the early fusion situation, more Logistic Regression coefficient feature importance scores with positive or negative values were found per modality, possibly indicating that some potentially relevant features are less hidden by noise when modelled in smaller batches. The highest metric value was the Maximum Criterion, which involved taking the best performing classifier of all, which was Modality 4 with a validation AUROC of 0.6340 ± 0.0152 and recall values of 0.68 for the normal label and 0.52 values for the Overweight or Obese label. In this way, a better performance than the early fusion approach was achieved.

### 3.3 Unsupervised multimodal pipelines

**Figure 7:**
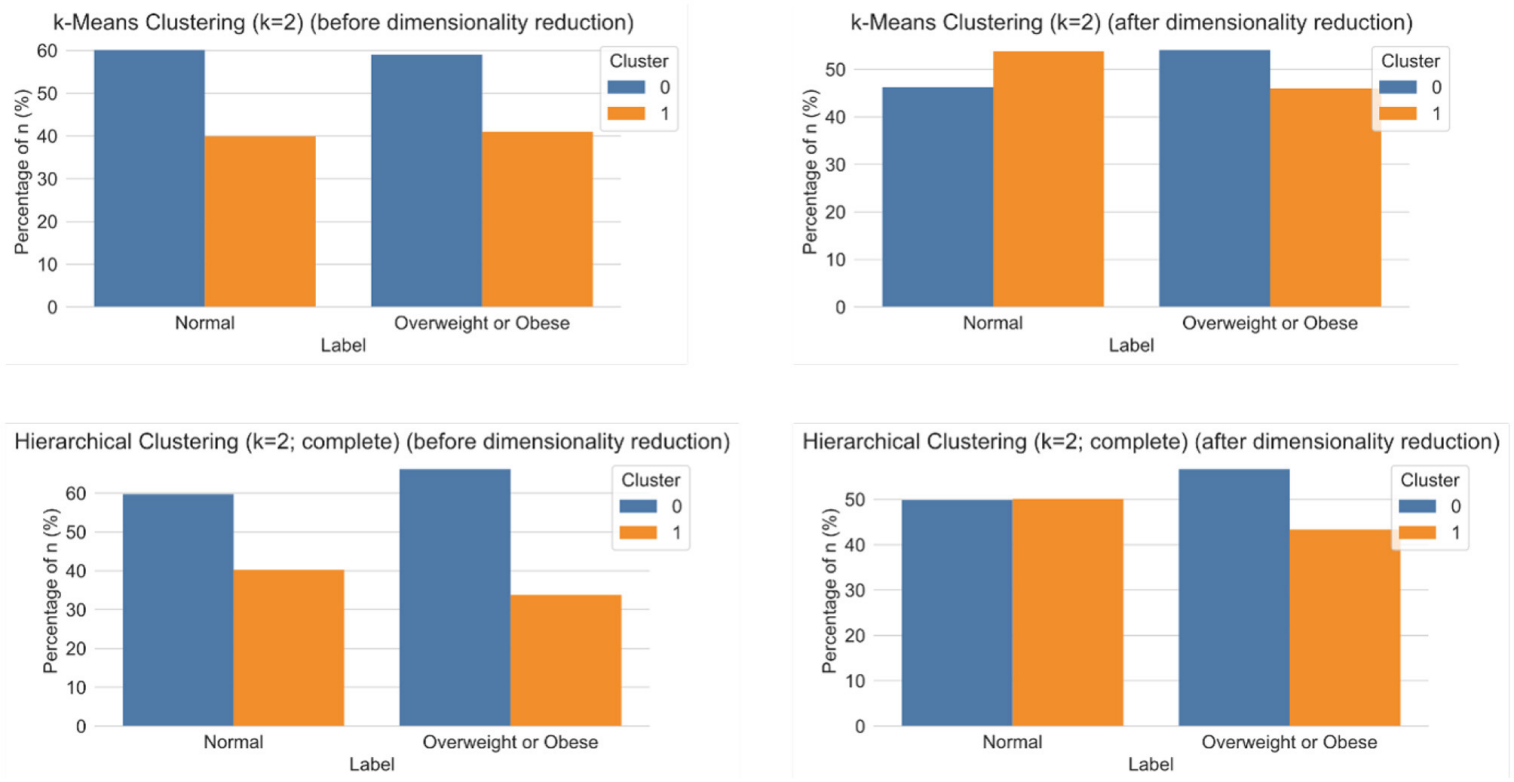
Results of clustering analyses for *k* = 2 using k-Means **(top)** and hierarchical clustering with complete distance method **(bottom)**, before **(left)** and after **(right)** dimensionality reduction.

**Figure 8:**
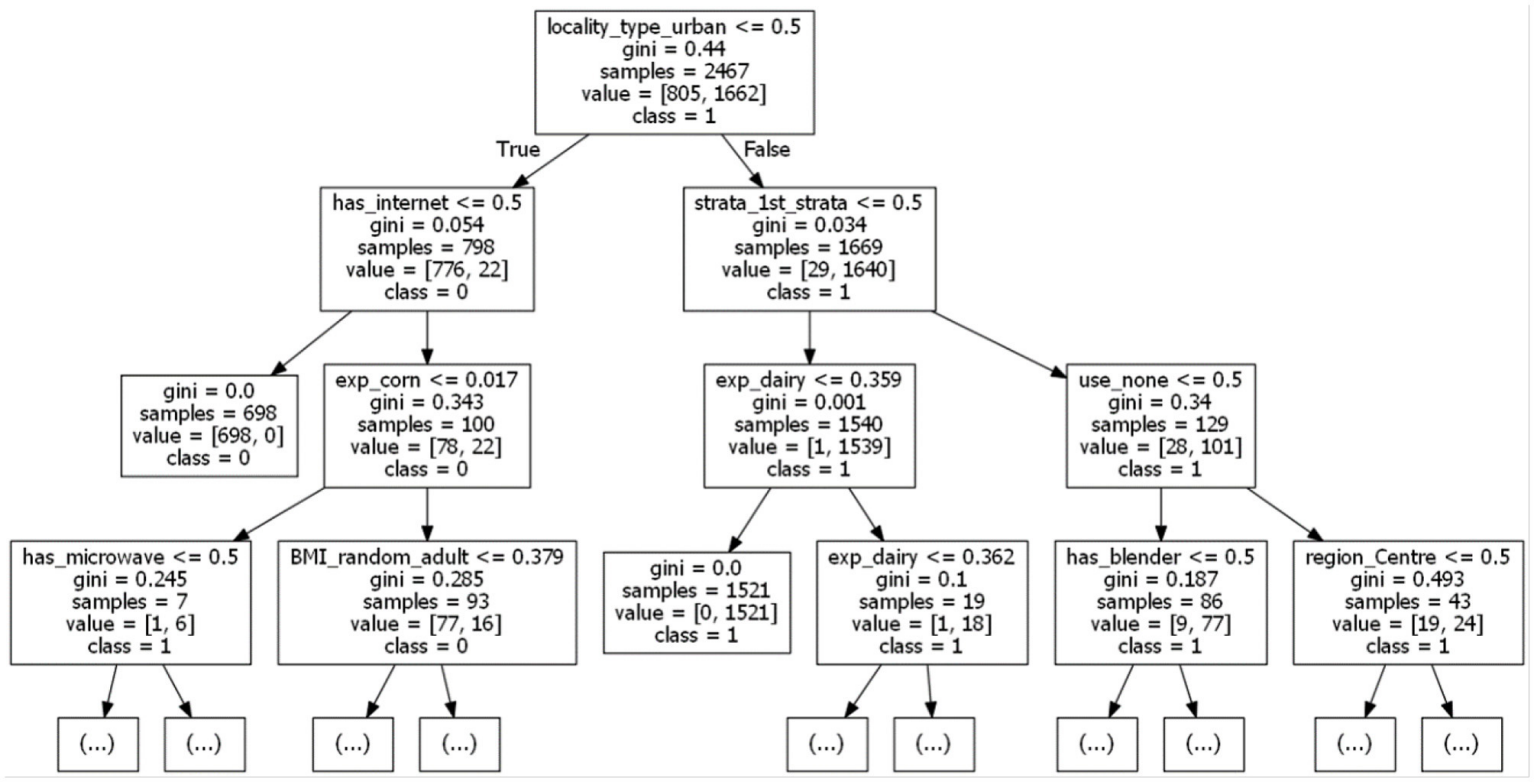
A relevant fragment of the decision tree used to interpret the clustering (*k* = 2 using k-Means, before dimensionality reduction).

**Figure 9:**
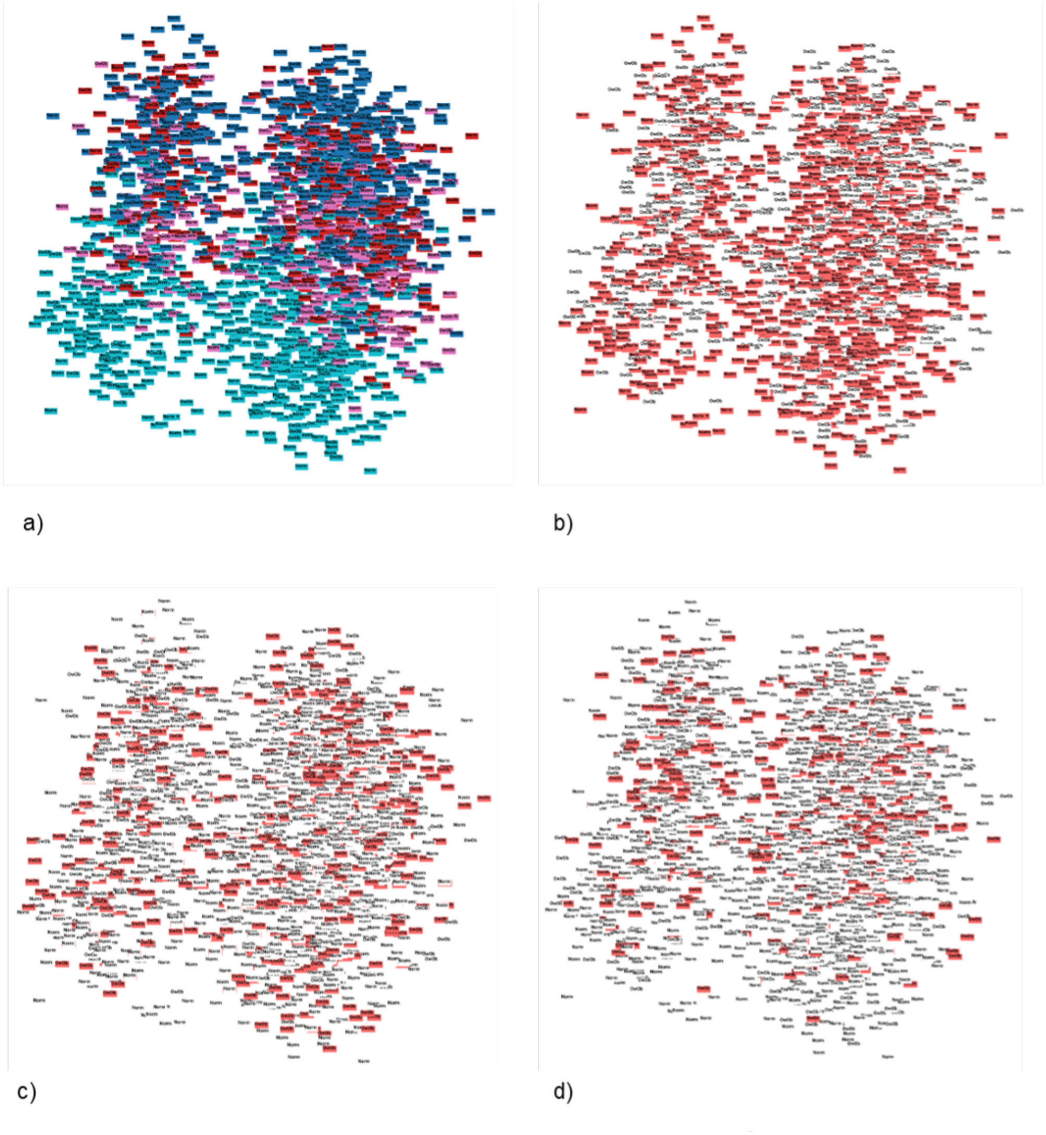
Patterns found using Principal Component Analysis (PCA) in 2D, constraining the search by the three-layered label and colouring by locality size. **(A)** PCA 2D label coloured by locality_size. **(B)** PCA 2D label coloured by locality_size with normal three-layered label marked. **(C)** PCA 2D label coloured by locality_size with overweight three-layered label marked. **(D)** PCA 2D label coloured by locality_size with obese three-layered label marked.

The multimodal dataset with encoded and normalised features was evaluated before and after dimensionality reduction (Figure 7). Although variability between clusters before and after dimensionality reduction decreased, clear patterns could still not be identified. Following Carbonell et al. (24), a decision tree was used to interpret the clusters in terms of underlying features (Figure 8). Note that the first three nodes are composed predominantly of home environment features. Looking at the first decision node, the instances that had urban locality type smaller than 0.5 ended up in the second cluster. The second node's features were having Internet and being in the first socio-economic stratum, while the third mostly had features about food expenses (corn and dairy) and a feature indicating not using the nutritional information on the front-packaging labels of processed food as buying advice. Three different dimensionality reduction techniques were employed: PCA, UMAP and t-SNE. Even if no clear pattern emerged when colouring by label, colouring by the ternary normal/overweight/obese instead of the binary used to train the supervised models produced results (Figure 9). A pattern can be seen for the smaller localities (< 2,500 inhabitants) where a considerably smaller number of children or adolescents suffering from overweight or obesity can be observed, a pattern that cannot be seen in any of the other locality sizes. Analogous results were obtained from UMAP and t-SNE (see Supplementary Figures S5, S6).

## 4 Discussion

### 4.1 Study findings

Our study found that integrally analysing the environment where the child or adolescent is growing up is essential to better understand the aetiology of paediatric obesity. Imagining a home environment built on some of the most important features identified, it is plausible to identify it as an obesogenic environment; Hobbs et al. (19) describe them as being characterised by surroundings, opportunities, or conditions of life, external to the individual, which promote overweight or obesity. Further studies should focus on adding features about behavioural patterns of the children and adolescents *per se*, analysing them in the context of obesogenic environments. Additionally, studies should investigate the daily habit patterns of adults that serve as behavioural examples for the paediatric population. Features to explore could include factors that overall contribute to having less time for building or maintaining healthy habits, such as exercise and leisure, the amount of time at work, the time spent commuting, the availability of spaces to exercise in, the safety situation in those spaces, and the economic and logistics accessibility of healthy foods. Finally, studies could be designed with other algorithms, other hyperparameters than the one we used (see Supplementary Figure S8), or ML methods in mind. In particular, larger samples could make possible the use of novel deep learning algorithms (41) to explore patterns even further.

After data pre-processing, 183 categorical and 28 numerical features had been extracted. Datasets were then prepared for each supervised and unsupervised multimodal ML pipeline, but the best performing late fusion approach did not outdo the best early fusion by much, as measured by AUROC. Rather than the quantitative predictive performance in itself, however, the truly meaningful aspect of the comparison was the analysis the differences of feature importance using the logistic regression coefficient (Figure 5). In the home environment modality, it was concluded that having a refrigerator, washing or drying machine, gas stove, a Mexico City dwelling, and belonging to the second socio-economic stratum were the top five most important features. In the *household income and expenses* modality, we were able to identify the expenses in dairy, bottled water, takeout or restaurant meals, tobacco, as well as the total food expense mattered as the most important. The third and fourth modalities concerned the adult randomly selected in each household and the former pointed to having an obesity diagnosis, having a father with high cholesterol or triglycerides, and having a meat intake of more than three days per week. The latter pointed to BMI, blood glucose, waist circumference, the number of minutes sitting down during the day, and the days per week of intense physical activity. By comparison, early fusion identified a total of eleven features as important, six of which were also present in the late fusion approach. The top five most important were BMI, having an obesity diagnosis, being single, seeking health care at private care, and having paid TV in the house. Significantly, early fusion results are incomplete, most likely because the selected features' interplay has a low signal-to-noise ratio. Therefore, considering results from two supervised approaches was beneficial.

### 4.2 Comparative study

Our study corroborates previous studies in finding that the lower prevalence of children suffering from obesity and adolescents in the lowest socio-economic stratum, majorly located in rural areas with < 2,500 inhabitants (42, 43). This prevalence has also been reported about children from households with high food insecurity, who on average weighed less than their peers from food-secure households. However, food insecurity and obesity/overweight did not appear to have a close correlation in our study. Another similar finding concerns the correlation of the child or adolescent's BMI with that of an adult in their immediate environment (15, 44). However, previous studies that also used ENSANUT data (43, 45) exclusively studied the correlation between maternal BMI classified as obesity, and children or adolescents with obesity. By contrast, our study found that any adult selected in the household having either a high BMI or the diagnosis of obesity were the strongest predictor in the population. Furthermore, the only kinship-related features appeared in Modality 5, and the most important feature concerned a “partner” relationship with the adult whose nutritional knowledge was evaluated. This finding echoes studies that found a positive correlation between overweight or obesity and female adolescents living with a partner (46). It is worth repeating that our study did not find the sex of the adult a relevant feature.

The multimodal ML analysis may help reduce cultural or social bias on the selection of specific features. We noticed a tendency towards studying almost exclusively maternal influence on paediatric obesity. In fact, a search in PubMed using the query “maternal” AND “paediatric obesity” yielded 1, 410 results, while “paternal” AND “paediatric obesity” yielded only 124 with all of them, without exception, used the term “paternal” to refer to both sexes and studied the relationship to risk factors of both parents. By contrast, studies focusing on obesogenic environments that built ML models for paediatric obesity prediction had a higher variety of features and included both sexes as equally relevant features, leaving the model to indicate importance.

Previous studies (32–34), while suggesting different numbers and types of dietary pattern, concurred that no pattern could be classified as healthy, since vegetables and fibre intake did not reach the minimum recommended portion, as stated by the WHO. Similarly, our study could not find a relevant pattern between the label and the consumption of food categorised as unhealthy. Some possible explanations of this phenomenon could be that self-reported surveys are affected by social pressure towards underreporting unhealthy and overreporting healthy food, or that the person being interviewed is unaware of their own eating habits. The most worrisome situation would be if both overreporting of healthy foods and underreporting of unhealthy foods were happening simultaneously.

Within the top ten most important features about the household, expenses in *takeout or restaurant meals* and in *soda* were identified. At the same time, other than a reduction of prevalence of children and adolescents suffering from obesity in the lowest socio-economic stratum, both overweight and obesity were undistinguishably spread among the second through fourth strata. This indicates that the choice to buy healthy vs. unhealthy food may not necessarily be related to accessibility, but rather linked to buying decisions. A study that investigated the price trends of healthy and unhealthy foods from 2011 to 2018 in Mexico corroborates this (47). Our study found that the expense in dairy was the most important feature for Modality 2 and appeared within the first three decision nodes used during the unsupervised traditional approach to explain the clustering decisions. From 1990 to 2004, some unfavourable changes happened with consequences persisting until 2018, including an increase in the price of grains and milk and a decrease of the price for sweet bread, cookies, and potato chips.

Comparing the study results to other studies that used ML to analyse paediatric obesity was a difficult endeavour. Only one study could be found that was built with data from Mexico (48). It used 16 anthropometric features to predict paediatric obesity in children from 6 to 13 years old from an indigenous community in Guerrero. Even though the model was considered to have good performance for identifying overweight and obesity, the sample consisted of only 221 children, and methods not recommended for small samples were used, with neither feature importance nor overfitting evaluated. Therefore, their conclusions are hard to compare with the present study. Hammond et al. performed a similar study in terms of using features from EHRs and publicly available data to build a model for predicting obesity (49). However, they did not study the Mexican population, their study population were under five years old, and only statistical feature associations, rather than feature importance of the ML models were presented. Neither studies that used multimodal ML to study paediatric obesity, nor used a combination of supervised and unsupervised methods were identified.

### 4.3 Implications for public health policy

One of the ongoing public health initiatives aims to promote long-term behavioural changes, focusing on improving the delivery of nutritional content information with new labels, to influence healthier buying decisions (11). A study in 2018 showed that most consumers did not use the new labels, however, because they had difficulties interpreting them (50). This finding concurs with our study. We showed that not using the nutritional information on the front of packaging to make a buying decision were within the first three decision nodes used during the unsupervised traditional approach to explain clustering decisions.

We have demonstrated that using multimodal ML methods could be worthy of consideration for developing public health policies, especially since the prevalence in Mexico has been increasing in the past decades regardless of the policies and strategies being implemented to control it, such as the 10% tax on sugar-sweetened beverages. Researchers concluded that the tax imposed is very low compared to the 66.9% for tobacco, which was effective in reducing the smoking behaviour of adolescents (51). The efficiency of using ML methods requires data in quality and quantity, which can be challenging to accomplish through sporadic efforts. Consequently, evolving the national health monitoring strategy towards a continuing monitoring health information system could help attain both enough data and data of sufficient quality. Furthermore, it could include multimodal data that could affect individuals seeking to develop and maintain healthy patterns. For instance, it could include health data reported by healthcare professionals, results of laboratory or diagnostic tests, information about working situation, social demographics, and other types of data relevant to individuals (cf. Supplementary Figure S7). Such a system could allow for continuous studies into obesogenic environments in Mexico.

In conclusion, our recommendations to change or improve the paediatric obesity situation include to transform the current periodic survey-based strategy to a continuous monitoring health information system, analyse other risk factors contributing to having less time for building or maintaining healthy habits, and explore the design of policies that may help families have healthy daily habits, such as investing in active public transportation. Our contribution to data-driven health informatics is that we present an alternative to analysing national information from a standardised survey, and that we seem to reach less biassed and more thoroughly analysed conclusions than previously reported from work without learning approaches. Finally, our work shows that practical use of multimodal ML for public health policy making is feasible and potentially leads to improving population health.

Three categories of limitations of our study must be noted, these are dataset-, sample-, and ML-related. First, self-reported information always comes with challenges with respect the credibility and quality of the data. The underlying study itself suffered from missing observations. We also have noted risks of over- as well as underreporting. Because the majority of articles selected in our scoping used traditional statistical methods for their feature selection, there is a risk of unwanted bias in our predictive features. To detect such bias, one may use fairness metrics to cheque whether the model's predictions are equitable across different stratifications of groups or features. An attractive bias removal strategy is testing with adversarial datasets to challenge model robustness. Secondly, the sample-related limitations include that obesity is so widespread in Mexico that it may bias the results to interpret them as causality. We also do not always differentiate between overweight and obesity. Some differences in factors that affect the development of paediatric obesity could be related to the age group of the subject. Thirdly, the distribution of the sample used was complex to determine, before we reached the conclusion that it was a mixed Gaussian. Principle component analysis assumes a linear relationship and re-expresses the data as linear combinations, hence it can skew non-linear relations. Analogously, the inertia parameter k-Means algorithm assumes that the clusters have a convex form and responds weirdly to elongated clusters.

These limitations notwithstanding, multimodal ML provided a comprehensive approach to analysing obesogenic environments. Our approach thus effectively presents an alternative approach to analysing national information from a standardised survey, which would seem to risk reaching biassed and less thorough conclusions.

## Data Availability

Publicly available datasets were analysed in this study. This data can be found here: ENSANUT: Encuesta Nacional de Salud y Nutrición (https://ensanut.insp.mx/encuestas/ensanut2018/index.php).
