# Supplementary material for: Multimodal machine learning for analysing multifactorial causes of disease—The case of childhood overweight and obesity in Mexico
Source: Front Public Health. 2025 Jan 7;12:1369041. doi: 10.3389/fpubh.2024.1369041 (PMC11752892; doi:10.3389/fpubh.2024.1369041)
Supplement: Supplementary file 1 [file Data_Sheet_1.pdf]

Supplementary Material

1 SCOPING REVIEW DETAILS

We performed a scoping review following the PRISMA guidelines, seeking to answer the question: *What knowledge about paediatric overweight or obesity has been elicited from ENSANUT data?* The scoping review allowed for a familiarization with variables present in the database that were previously studied in connection with paediatric overweight or obesity, and it enabled conducting an informed feature extraction and modality definition. The search included MESH terms and synonyms for three search terms: Paediatric Obesity, ENSANUT and Mexico. The PRISMA flow diagram of the scoping review is available in Figure S1. The initial search found 118 articles in 7 different databases, from which duplicated papers (n=68) and papers whose titles indicated an explicit irrelevance towards the research question (n=11) were removed. Out of the 39 articles included in the abstract screening, the ones that did not use ENSANUT as primary source (n=9) and the ones that only used a National Health and Nutrition Survey from a country other than Mexico (n=8) were removed. Only 22 were part of the full-text screening, from which one was removed because ENSANUT was not used as a primary source, leaving a total of 21 articles, which corresponded to 21 different studies. The PubMed search query was:

(Pediatric Obesity[Mesh] OR ”Pediatric obesity” OR ”obesidad pediatrica” OR ”Obesity in Childhood” OR ”obesidad en la niñez” OR ”obesidad en escolares” OR ”obesidad en edad escolar” OR ”Childhood Onset Obesity” OR ”Child Obesity” OR ”obesidad en niñ*” OR ”Childhood Obesity” OR ”obesidad de la niñez” OR ”Adolescent Obesity” OR ”obesidad adolescente” OR ”Obesity in Adolescence” OR ”obesidad en adolescentes” OR ”obesidad en la adolescencia” OR ”Infantile Obesity” OR ”obesidad infantil” OR ”Infant Obesity” OR ”obesidad en infantes” OR ”Childhood Overweight” OR ”sobrepeso en la infancia” OR ”sobrepeso en la niñez” OR ”sobrepeso en escolares” OR ”sobrepeso en edad escolar” OR ”Infant Overweight” OR ”sobrepeso infantil” OR ”sobrepeso en niñ*” OR ”Adolescent Overweight” OR ”sobrepeso en adolescentes” OR ”sobrepeso adolescente” OR ”sobrepeso en la adolescencia”) AND (”ENSANUT” OR ”Encuesta Nacional de Salud y Nutrición” OR ”National Survey on Health and Nutrition” OR ”Mexican National Survey on Health and Nutrition” OR ”National Survey on Health and Nutrition in Mexico” OR ”National Health and Nutrition Survey” OR ”Mexican National Health and Nutrition Survey” OR ”National Health and Nutrition Survey in Mexico” OR ”National Health and Nutrition Survey of Mexico”) AND (”Mexi*” OR ”México”)

while the search query for the other repositories were:

(”Pediatric obesity” OR ”obesidad pediatrica” OR ”Obesity in Childhood” OR ”obesidad en la niñez” OR ”obesidad en escolares” OR ”obesidad en edad escolar” OR ”Childhood Onset Obesity” OR ”Child Obesity” OR ”obesidad en niñ*” OR ”Childhood Obesity” OR ”obesidad de la niñez” OR ”Adolescent Obesity” OR ”obesidad adolescente” OR ”Obesity in Adolescence” OR ”obesidad en adolescentes” OR ”obesidad en la adolescencia” OR ”Infantile Obesity” OR ”obesidad infantil” OR ”Infant Obesity” OR ”obesidad en infantes” OR ”Childhood Overweight” OR ”sobrepeso en la infancia” OR ”sobrepeso en la niñez” OR ”sobrepeso en escolares” OR ”sobrepeso en edad escolar” OR ”Infant Overweight” OR ”sobrepeso infantil” OR ”sobrepeso en niñ*” OR ”Adolescent Overweight” OR ”sobrepeso en adolescentes” OR ”sobrepeso adolescente” OR ”sobrepeso en la adolescencia”) AND (”ENSANUT” OR ”Encuesta Nacional de Salud y

Nutrición" OR "National Survey on Health and Nutrition" OR "Mexican National Survey on Health and Nutrition" OR "National Survey on Health and Nutrition in Mexico" OR "National Health and Nutrition Survey" OR "Mexican National Health and Nutrition Survey" OR "National Health and Nutrition Survey in Mexico" OR "National Health and Nutrition Survey of Mexico") AND ("Mexi*" OR "México")

2 DATA PREPARATION

The data in the ENSANUT-18 database comes with a methodology designed to have state, regional and national representation for rural and urban strata (Shamah-Levy et al., 2019). Randomised households are selected to provide information about access to health services, family factors, socio-economic factors, diet and exercise habits, anthropometric measurements, and biological tests (complete blood counts and micronutrients). Informed consent is required for each household that participates in the survey, with the format being previously approved by the Ethics Committee of the National Institute of Public Health of Mexico. Since 1999, all survey results have been digitalised, de-identified and made publicly available for commercial and scientific use. ENSANUT-2018 data was collected between July 2018 and June 2019 by a team of general interviewers and specialists (nutritionists and nurses). The 18 questionnaires consist of 788 questions in total. The questionnaires are divided into two components: health and nutrition, as well as four themes: home level, individual health, nursing, and nutrition. ENSANUT data is built by selecting randomised homes to provide the information: 50,000 homes for the health component and 32,000 homes for the nutrition component. A set of unique identifiers that work together as a multi-level key, for the home, household, and individual, are used to register the information in the database.

A set of unique identifiers that work together as a multi-level key was used to register the information in the database. For instance, to identify the city block where the house selected was located, the variable UPM was given a unique number. In the same way, unique numbering was given to the houses (VIV_SEL) within the blocks, the households within a house (HOGAR) and each resident within a household (NUMREN). Hence, the concatenation of all unique numbers serves the purpose of identifying each level uniquely. Following the example in Figure 4, the unique identifiers of the people on the right would be 10_7_1_1 to 10_7_1_4. In the same way, the identifier of their household would be 10_7_1, their house 10_7, and their city block 10.

Such keys facilitated the correlation of the information about the children or adolescents selected for the study, hereon referred to as our study population, with that of their families or dwellings. The information collected from the questionnaires was organised in 35 Comma-Separated Value (CSV) files linked by the unique identifiers. Each CSV has its own variable catalogue describing the questionnaire it was obtained from, variables obtained from the questionnaire items, original questions asked, data types of each variable, answer translation for categorical variables, valid formats per answer, and internal codes used. The CSV catalogues were used to analyse and select the features to include in each modality. After selecting the features, each dataset was concatenated based on the unique keys of the study population. Depending on the modality, the dwelling, household, or personal keys were used for the concatenation.

The content of the surveys has been adjusted throughout the years in accordance with the knowledge gaps identified on the previous instances and to assess the efficacy of policies and strategies. For instance, since the results of ENSANUT 2012 and 2016 indicated that the prevalence of non-communicable diseases increased greatly despite a strategic modification to the regulation on the labelling of processed food and non-alcoholic beverages that aimed to make it clearer to the population, a new survey was added to ENSANUT 2018. This new survey included a knowledge test on the nutritional information contained in

the front-packaging. When the knowledge gap on nutritional information was detected, the contents of this regulation were again modified to adapt to the population's needs. Nevertheless, the knowledge used as basis for public health strategies has been obtained using only mathematical and statistical analysis associations and correlations. Hence, it is plausible that other methods such as those of Machine Learning (ML) could provide additional knowledge on the predictors of paediatric overweight and obesity in Mexico, thanks to its capacity to analyse complex and non-linear patterns.

3 PRE-PROCESSING DETAILS

The required step after the feature selection and modality definition was to extract the metadata of the sample of the study population, which included: the unique identifiers for house (house_ID), household (household_ID), person (person_ID), the region in Mexico were the subject lived (region), the socioeconomic strata (strata), the age in months (age_months), the sex (sex), the number of standard deviations for the BMI-for-age z-score (BMI_SD), the label in a categorical format (label_cat), and the label as integer (label). The population from 5 to 19 years was filtered down in accordance with the age in months (60 to 228 months), so that it resembled the BMI-for-age z-score tables defined by WHO. Data from pregnant and lactating adolescents was dropped as well as missing or "not registered" values according to the CSV catalogue. Afterwards the average weight and height were computed as the average of the first and second observation of each parameter, which was required for the BMI-for-age z-scores calculation. After running the functions and plotting the distribution, an unrealistic left and right tail were identified (left: -12.5SD, right: +7.5SD). The recommendation of the WHO for keeping valid thresholds is to limit the SD to values between -3SD and +3SD (De Onis, 2017), however, previous studies and official reports of ENSANUT data [33,91], used a range between -5SD and +5SD. Hence, to be able to compare the results of this study with the studies found during the Scoping review, values between -5SD and +5SD were kept. This strategy left $n = 11320$ with a Gaussian distribution (Figure S4, top). However, while analysing the class balance, the categorical label was noted as deficient. The Underweight label was dropped and Overweight was fused with Obese to see if this strategy gave a better label balance. Since the binary label improved the class balance and the observations were sufficient ($n = 10301$), it was decided to keep the label binary. Worth noting is that the binary label distribution is non-Gaussian with a right tail, hence, the upcoming feature scaling strategy should be standardization rather than normalization (Figure S4). However, when analysing the plotted distributions of the numerical features it was discovered that the dataset distribution was mixed Gaussian instead, meaning that some features had Gaussian distributions, such as average systolic and diastolic blood pressures, while others had right or left tails, such as blood glucose and haemoglobin values. Finally, the demographic metadata of the study population is shown in Figure S7.

While the iterative data pre-processing phase meant a different process for each modality, all contained similar steps during the feature extraction, exploratory data analysis, feature engineering, data cleaning, and feature scaling. For instance, given that the question ID of the features in the original catalogues was repeated in several questionnaires, a new, unique and more descriptive feature name was introduced. During the analysis, Pearson correlations were plotted in a heatmap to identify features that could cause multi-collinearity [98]. For example, for two categorical features x = *type of stove* and y = *type of fuel used for the stove*, x and y will always have a linear relationship. Multi-collinear features were dropped to avoid noise or bias in the models. Data cleaning always concerned first identifying the values or codes used in each CSV in the database to represent missing values, which was a challenge because it varied in each CSV, and later dropping columns that had more than 35% missing values. The only occasion in which that rule was adapted was for the unimodal preparation of the dataset, where a threshold of 30% was preferred

to keep the minimum representative size of $n \geq 2344$. Finally, feature scaling meant either the creation of dummy variables or the standardization of the features.

4 COMPUTATIONAL COMPLEXITY OF THE CLASSIFIERS EMPLOYED

For analysis of worst-case computational complexity, it is necessary to consider both training and prediction (inference) phases. The Linear Classifier with Elastic Net Regularization has a training complexity reflecting the need to solve the regularised optimisation problem, which typically involves iterative methods like coordinate descent. Its prediction complexity is a multiple of the sample size, as it requires computing the dot product of the input features with the learned coefficients.

For k-Nearest Neighbour, the training complexity is constant, as k-NN is a non-parametric method that involves storing the training data without any explicit training phase. For prediction, the algorithm needs to compute the distance between the test sample and all training samples to find the nearest neighbours.

The training complexity of Decision Tree is dependent on sample size, number of features, and the logarithm of the sample size, as constructing the tree involves sorting the data multiple times and splitting nodes, which scales logarithmically with the number of samples. Its prediction complexity is is only the last of the three factors, assuming a balanced tree. The complexity arises from traversing the tree from the root to a leaf node.

Finally, Random Forest training complexity depends on the number of trees in the forest. This is because each tree in the forest is trained independently, similar to a decision tree, but with additional overhead for feature subsampling and bootstrapping. The prediction complexity requires each sample to be classified by all trees in the forest, and the results are aggregated. We have summarised all of the above in Figure S9.

5 STATISTICAL DETAILS

The transformations made as part of feature engineering, for each of the five modalities, are detailed in Figure S10. Hyperparameter tuning values, for each classifier, are in Figure S11. Drops due to feature engineering are detailed in Figure S12 and S13. Finally, the late fusion meta-classifier details are in Figure S14.

REFERENCES

Shamah-Levy T, Romero-Martínez M, Cuevas-Nasu L, Méndez Gómez-Humaran I, Antonio Avila-Arcos M, Rivera-Dommarco JA. The mexican national health and nutrition survey as a basis for public policy planning: Overweight and obesity. *Nutrients* **11** (2019) 1727.

De Onis M. World Health Organization child growth standards. *The Biology of the First 1,000 Days* (2017) 17–32.

Carbonell MF, Boman M, Laukka P. Comparing supervised and unsupervised approaches to multimodal emotion recognition. *PeerJ Computer Science* **7** (2021) e804.

SUPPLEMENTARY TABLES AND FIGURES

Figures

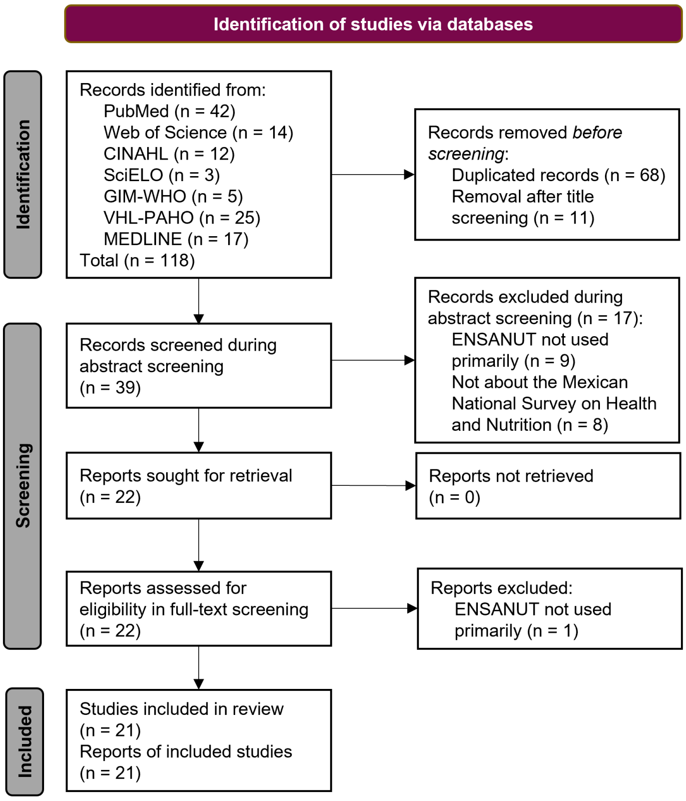

Figure S1. Preferred Reporting Items for Systematic Reviews and Meta-Analyses (PRISMA) flowchart for the scoping review.

Category	Observations
Dietary patterns (DP)	• The article that analysed the highest number of variables out of all reviewed was in this category and analysed 45.<br>• None of the articles that categorized DP found a healthy DP due to the generalised low intake of fruits, vegetables, and fibre.<br>• All the studies that grouped DP were based on the Food Frequency Intake (FFI) questionnaire used in ENSANUT 2006, 2012, 2016, and 2018.<br>• Most studies concluded that the FFI questionnaire presented generally overreporting of healthy foods and underreporting of unhealthy foods, especially so in the cases were the person answering the questionnaire had OW/OB.
Environment	Relevant sociodemographic variables to include are region were the subject lives, type of locality (rural or urban), size of locality, Socio Economic Strata (SES), enrolment in governmental food aid programs, and the result from level of Food Security obtained from the Latin American and the Caribbean Food Security Survey (ELCSA for its abbreviation in Spanish: "Encuesta Latinoamericana y del Caribe de Seguridad Alimentaria").
Epidemiology	• Descriptive statistics and logistic regression models are the most common statistical analysis strategies used with the database.<br>• One study proposed a stratified approach of interpreting reduced or excess weight, however, it was more beneficial to identify the causes of children with an underweight status than for those with excess weight.
Family dynamics	Only found studies that analysed the relationship of a social factor with maternal-characteristics (professional situation or breastfeeding practices).

Figure S2. Main observations from the scoping review per category.

Risk factor category	Author (year)	Research aim	Study population	Variables (source)	Type of analysis	Conclusions/Findings
Dietary patterns (DP)	Afeiche et al (2018) [17]	Compare sugar intake in children in China, Mexico, and U.S., and identify the main sources.	Children 4 to 13 years old (n = 3,980 Mexico)	3 (ENSANUT 2012)	Exploratory Data Analysis and Student's T-test to compare mean statistical significance between countries.	Compared to Chinese children, mean intakes of added sugars were 6 and 8 times higher among Mexican children. Sugary beverages contributed around 31% and 43% of total sugar intakes in Mexican children.
	Arango-Angarita et al (2019) [15]	Analyse the association between Dietary Energy Density (DED) and OW/OB.	Adolescents 12 to 19 years old (n = 2,203)	13 (ENSANUT 2012)	Bivariate and multivariate statistical models employing Stata's SVY module for complex samples.	Positive association between high DED and BMI-for-age z-score, but no significant association with OW/OB.
	García-Chávez et al (2020) [91]	Analyze the association between DP and OW/OB.	Children 5 and 11 years old (n = 2,751)	42 (ENSANUT 2012)	Descriptive statistics. Bonferroni association analysis using a Poisson regression model.	Four DP were identified: traditional, industrialized, varied, and modern. No DP could be defined as healthy.
	Jiménez-Aguilar et al (2014) [92]	Compare the current consumption of fruits and vegetables, with the international recommendations.	Children, aged 6 to 12 years (n = 1,506)	10 (ENSANUT 2012)	Linear regressions, bivariate logistic model regression. Multivariate logistic regression model.	Only 3 out of every 10 children aged 6-12 years (34.4%) meet the international recommendations for consumption of fruits and vegetables.
	Rodriguez-Ramírez et al (2011) [16]	Identify DP and determine their association with being OW/OB.	Children 5 to 11 years of age (n = 8,252)	27 (ENSANUT 2006)	k-means clustering for the DP group definition. Linear and logistic regression models for association with independent variable.	Five DP were identified: rural, diverse, western, whole-milk and sweet (WMS) and sweet cereal and corn dishes (SCCD). No DP could be labelled as healthy. WMS and SCCD had the highest correlation with OW/OB.
	Zárate-Ortiz (2019) [18]	Describe dietary patterns of Mexican adolescents and to associate these with OB/OB, anemia, and stunting.	Adolescents 12 to 19 years old (n = 7380)	35 (ENSANUT 2006)	Principal Component Analysis (PCA) plus descriptive statistics and Cox regression analysis.	Four DPs were identified: nontraditional and breakfast-type, Western, plant-based, and protein-rich. Prevalence of OW/OB higher in Western and plant-based DP.
	García-Chávez et al (2018) [21]	Identify the DP of Mexican schoolchildren and to assess their associations with sociodemographic factors.	Children 5 and 11 years old (n = 2,751)	40 (ENSANUT 2012)	Descriptive statistics, comparisons using Bonferroni method and association analysis using Poisson regression model. K-means clustering for defining the DP.	Four dietary patterns were identified: traditional, industrialized, varied, and modern. 'Industrialized' pattern tended to be consumed more by children in middle and high socioeconomic strata, while the 'Modern' pattern was most associated with urban areas; and the strongest association was observed for this 'Modern' DP and non-indigenous population.
	Medina-Zacarías et al (2020) [90]	Identify sociodemographic and dietary patterns associated to OW/OB in female adolescents.	Female adolescents 12 to 19 years old (n = 1,072)	45 (ENSANUT MC 2016)	Logistic regression models.	OW/OB has a positive correlation with living with an adult more than 20 years old with excess weight, living with a partner, dedicating to household work while simultaneously working in an urban setting, living in the South region of Mexico, having a high welfare condition index, being more than 2 hours in front of a screen, and a high energy intake.
Family dynamics	Espinosa et al (2018) [22]	Evaluate the impact of extra-domestic maternal professional participation in the excess weight of scholar-age population.	Children 6 to 11 years (n = 14,718)	10 (ENSANUT 2012)	Statistic logistic, binomial, bivariate and multivariate models.	Extra-domestic maternal professional participation was not an explicative factor for excess weight in children when household characteristics were considered. A pattern was found between children with excess weight and those who lived with a family member other than their parents. Authors hypothesized that non-parental family members facilitated unhealthy food consumption.
	Campos et al (2021) [25]	Examine breastfeeding, individual and household risk factors for malnutrition.	Children 6 to 35 months (n = 2,089)	13 (ENSANUT 2012)	Logistic regression models. Statistical comparative analyses (frequencies, weighted and unweighted percentages and means) for set/subset representativeness.	No evidence for a protective effect of breastfeeding on child overweight. Shared risk factors for child overweight and stunting among Mexican and Mexican Americans were high- and low-birthweight, correspondingly.
Environment (physical, social, cultural, economic, or political)	Arango-Angarita et al (2022) [15]	Estimate the association between store density and OW/OB, with sugar-sweetened beverages (SSB) consumption as a mediator.	Adolescents 12 to 19 years old (n = 2,637)	10 (ENSANUT MC 2016)	Quantile regression models to obtain median SSB consumption. Bonferroni adjustment. Chi-square test. Generalized Structural Equations Models (SEM) to establish direct associations between store density and SSB consumption.	Consumption of SSBs in adolescents with OW/OB was higher than in those with normal weight (258.6 vs. 215.7 mL/day, respectively) and in adolescents in areas with lower SSB prices (244.8 mL/day).
	Basto-Abreu et al (2019) [28]	Estimate the ten-year impact on health outcomes and quality of life years as well as the cost-effectiveness of SSB tax.	Population ages 2 to 100 years (n = NA)	7 (ENSANUT 2012 and 2016)	Mathematical modelling simulation. Probabilistic sensitivity analyses.	Over ten years the excise tax on sugar-sweetened beverages implemented in Mexico would lead to 239,900 fewer cases of obesity, as well as meaningful reductions in obesity-related disease and health care costs and increases in healthy life.
	Illescas-Zárate et al (2021) [93]	Estimate the potential effect of the non-essential energy-dense foods (NEDF) tax on BMI and the prevalence of OW-OB in Mexican children.	Children and adolescents 6 to 17 years old (n = 3,912)	Not available (ENSANUT 2012)	Mathematical model based on Dynamic Childhood Growth and Obesity Model (DCGO), PCA to identify demographic covariates for the model.	Observed reductions in NEDF consumption after the tax should result in a national average reduction in energy consumption of 17.4 kcal/person/day and a weight difference of −0.40 kg, −0.39 kg/m2 in BMI, and a −1.7 and a −0.4 pp in the prevalence of overweight and obesity at the end of the first year with the tax.
	Torres-Álvarez et al (2020) [2]	Estimate the potential impact of Mexico's SSBs tax in children and adolescents OW/OB.	Children and adolescents 5 to 18 years old (n = 5,513)	3 (ENSANUT 2012)	Mathematical model based on Dynamic Childhood Growth and Obesity Model (DCGO).	Suggests that the current SSBs tax could represent an effective national policy to reduce body weight in children and adolescents. For high SSBs consumers, which represent 61.5% of the total population, the current 10% tax would produce sizable body weight reductions.
	Quezada-Sánchez et al (2020) [94]	Estimate the malnutrition prevalence, describe the prevalence variability among municipalities, and document the coverage of governmental aid of the municipalities with higher malnutrition prevalence.	Children 0 to 59 months of age (n = 9,441)	3 (ENSANUT 2012)	Logistic regression with a Normalized Generalized Model.	Short stature and low weight were heterogeneous among the municipalities, while OW/OB were homogeneous. Governmental aid was missing in around 50% of the municipalities with high indices of short stature and low weight.
	Shamah-Levy et al (2017) [27]	Examine the association between High Foof Insecurity (HFI) and risk of childhood stunting and to determine whether this association is modified by maternal–child OW/OB.	Mothers and their children from 1 to 11 years old (n = 5,087 mothers and n = 7,181 children)	8 (ENSANUT 2012)	Descriptive statistics. Logistic regression models.	Children from HFI households weighed less than their peers from food-secure households. Moderate and severe HFI were associated with low height in children who were under 5 years old and lived with mothers with overweight or obesity.
Epidemiology	Selem-Solis et al (2018) [95]	Propose a new method to interpret weight in children and adolescents based on a combination of BMI-for-age and Height-for-age z-scores from WHO.	Children and adolescents 0 to 19 years old (n = 41,001)	2 (ENSANUT 2012)	Conditional probability calculation, comparison of each value against the expected in a normal distribution.	The "Nutrimetry" method proposed offer more specific stratification than WHO's BMI-for-age z-scores, allowing a better population description and analysis for the underweight or underdeveloped children or adolescents.
	Shamah-Levy et al (2018) [96]	Update the OW/OB prevalence in Mexican population under 20 years.	Children and adolescents 0 to19 years old (n = 7,758)	3 (ENSANUT MC 2016)	Descriptive statistics and logistic regression models.	No substantial increase in prevalence of OW/OB was observed from 2012 to 2016. However, a 3.6% increase in OW/OB was observed in rural populations while in urban popula-tions OW/OB decreased by 5%.
	Shamah-Levy et al (2019) [31]	Show prevalence of OW/OB in Mexican population and examples where ENSANUT was used for public policy.	All Mexican population (n = NA)	4 (ENSANUT 2006, 2012, 2016)	Descriptive statistics. Logistic regression models.	Significant association between obesity in adult women and households that suffer severe food insecurity when compared with those women without food insecurity.
	Shamah-Levy et al (2020)	Estimate the prevalence of OW/OB.	Children and adolescents 0 to19 years old (n = 44,069)	10 (ENSANUT 2018)	Descriptive statistics. Logistic regression models.	Positive correlation between maternal BMI (obesity) and children or adolescent with obesity. Inverse correlation between fiber intake and OW/OB.
	Shamah-Levy et al (2019) [97]	Describe the prevalence and trends of OW/OB vulnerable populations and localities with <100k inhabitants.	Children, adolescents, and their parents (n = NA)	8 (ENSANUT 2012 and 2018)	Descriptive statistics focusing on comparing results.	In scholar children without food aid programs, obesity increased 97% between 2012 and 2018.

Figure S3. Results of literature review on studies that use ENSANUT data as primary source.

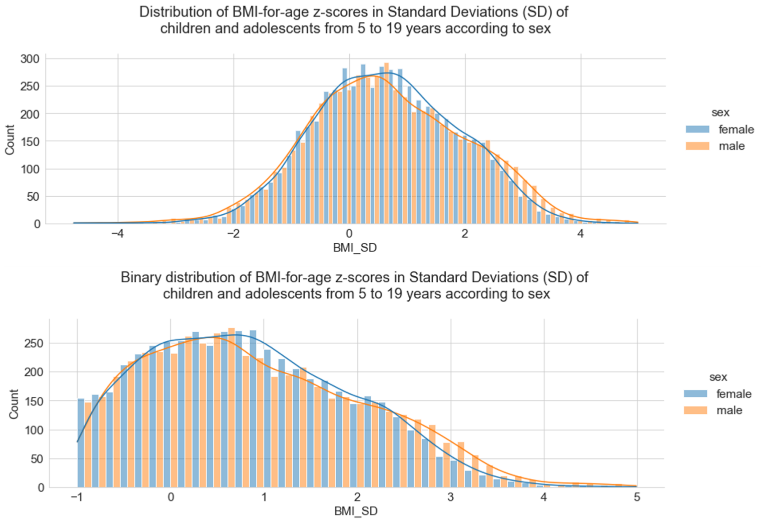

Figure S4. Comparison of the BMI-for-age z-score SD distributions by sex, when using a categorical label (top) versus a binary label (bottom).

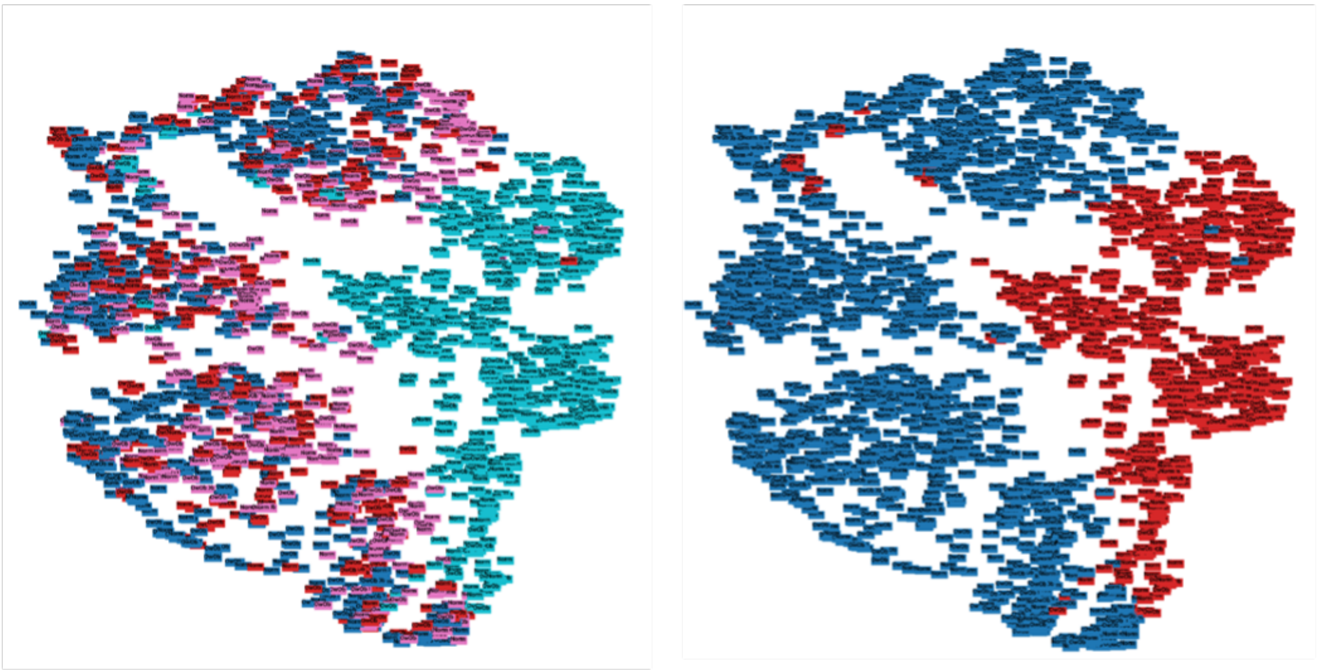

a) UMAP 2D label colored by locality_size   b) UMAP 2D label colored by locality_type

Figure S5. The Uniform Manifold Approximation and Projection (UMAP) algorithm ran for 500 epochs, a fixed parameter for what the algorithm considers to be small data sets, and 36 neighbours. Four to five clusters could be observed without any identifiable pattern when colouring it by label. Nevertheless, when colouring by locality size and type, at least one pattern can be identified, as for the PCA algorithm.

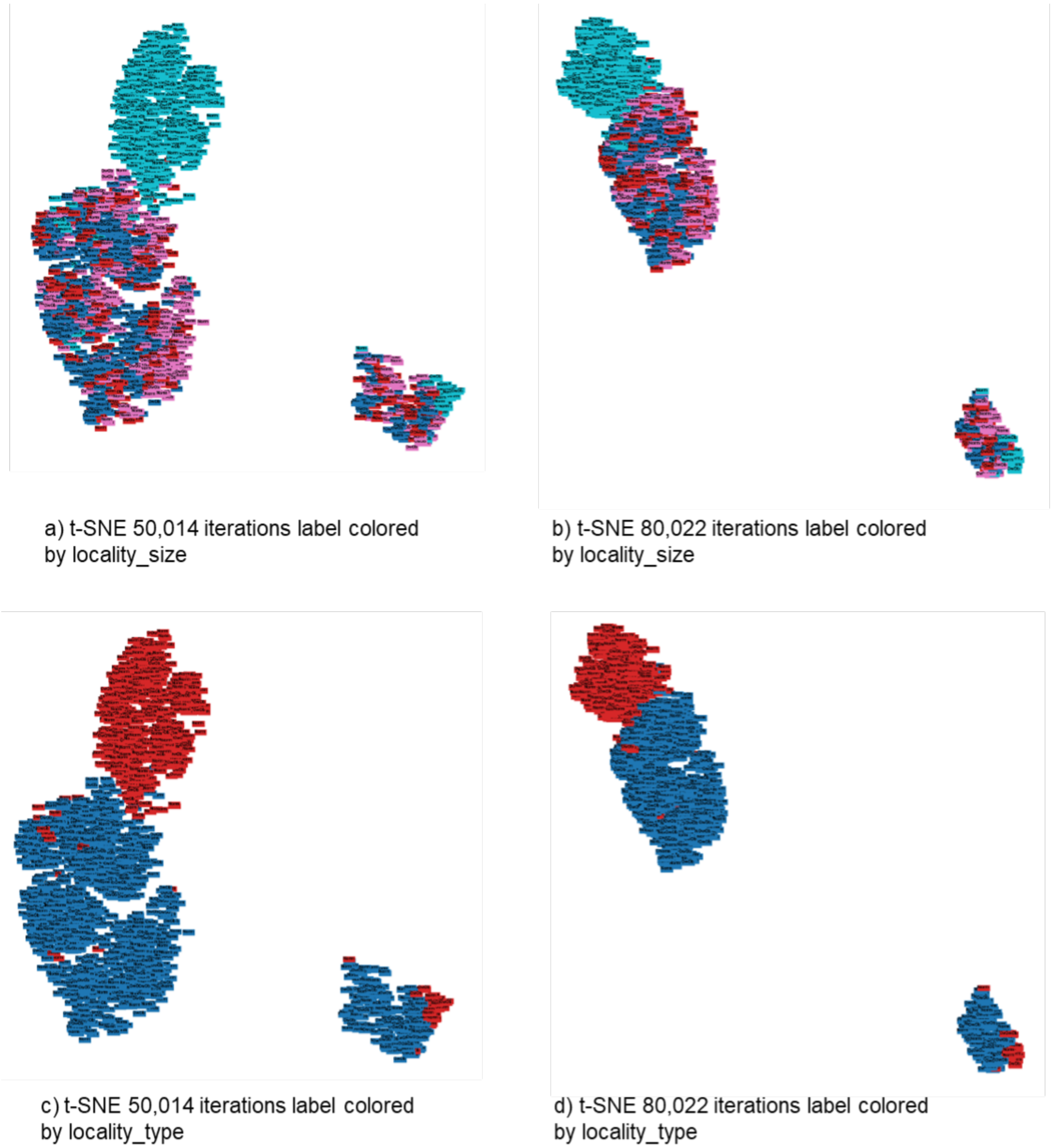

Figure S6. The t-distributed Stochastic Neighbor Embedding (t-SNE) ran for 50014 and for 80022 iterations until no changes in convergence were noticeable (bottom). The same parameters as (Carbonell et al., 2021) were entered (perplexity: 25 and learning rate: 10). Again, the same pattern emerged as for the PCA.

Data	Percentage
Age	
5	7.9%
6	8.5%
7	8.4%
8	8.7%
9	8.6%
10	6.9%
11	7.1%
12	6.6%
13	6.7%
14	6.4%
15	6.5%
16	5.7%
17	6.0%
18	5.6%
19	0.4%
Sex	
Female	50.2%
Male	49.8%

Data	Percentage
Socioeconomic Strata	
1st (lowest)	28.8%
2nd	55.0%
3rd	12.4%
4th (highest)	3.8%
Region	
Centre	36.4%
South	20.0%
North	41.0%
Mexico City	2.6%
Locality size	
< 2,500	36.7%
2,500-14,999	19.2%
15,000-99,999	15.3%
>100,000	28.8%
Locality type	
Urban	63.4%
Rural	36.6%

Demographic features of the random adult in Modality 3

Data	Percentage
Age group	
20-29	19.1%
30-39	35.3%
40-49	27.0%
50-59	10.5%
60-69	5.1%
70-79	2.0%
80-89	0.9%
≥ 90	0.2%
Sex	
Female / Male	57.6% 42.4%

Data	Percentage
Kinship	
Parent	68.7%
Grandparent	10.8%
Parent or uncle/aunt	5.6%
Sibling	5.5%
Unknown	3.7%
No kinship	1.9%
Mother/father or uncle/aunt	1.7%
Sibling or cousin	0.4%
Partner	0.3%
Mother/father-in-law	0.3%
Great grandparent	0.1%
Sibling or brother/sister-in-law	<0.0%
Other	<0.0%

Demographic features of the adult overseeing cooking in the household in Modality 5

Data	Percentage
Age group	
20-29	24.3%
30-39	39.5%
40-49	25.6%
50-59	7.5%
60-69	2.3%
70-79	0.6%
80-89	0.3%
≥ 90	0.0%
Sex	
Female / Male	58.7% 41.3%

Data	Percentage
Kinship	
Parent	70.5%
Sibling	7.9%
Grandparent	6.6%
Parent or uncle/aunt	5.3%
Unknown	3.8%
Mother/father or uncle/aunt	1.9%
No kinship	1.9%
Parent or brother/sister-in-law	1.1%
Sibling or cousin	0.4%
Partner	0.3%
Great grandparent	0.1%
Mother/father-in-law	0.1%
Sibling or brother/sister-in-law	0.0%

Figure S7. Demographic metadata of the study population.

Classifier	Hyperparameter	Series
Elastic Net	alpha	[0.0001, 0.00025, 0.0005, 0.00075, 0.001, 0.005, 0.0025, 0.0075, 0.01, 0.05, 0.1, 1]
	n_iter_no_change	[20, 50, 100, 150]
	l1_ratio	[0.049, 0.10, 0.15, 0.20, 0.25, 0.30, 0.35, 0.40, 0.45, 0.50, 0.55, 0.60, 0.65, 0.70, 0.75, 0.80, 0.85, 0.89, 0.95, 0.99]
	penalty	[‘elasticnet’]
	class_weight	[‘balanced’]
	loss	[‘log’]
	max_iter	[2500]
	random_state	Seed = 27
k-NN	n_neigh	Start-stop ranges from 1 to 905 on 5-sized steps (e.g. 1 to 5, 10 to 15, and such).
	leaf_size	[10, 20, 30]
	algorithm	['ball_tree','brute', 'kd_tree']
	weights	['uniform'],
	metric	['manhattan']
	n_jobs	[-1]
Decision Tree	criterion	['gini', 'entropy']
	splitter	['best', 'random']
	max_depth	Start-stop ranges from 5 to 55 on 5-sized steps (e.g. 5 to 10, 15 to 20, and such).
	min_samples_split	[0.1, 0.2, 0.3, 0.4, 0.5, 0.6, 0.7, 0.8, 0.9, 1.0]
	min_samples_leaf	[0.1, 0.2, 0.3, 0.4, 0.5]
	max_features	Number of max features in the given dataset.
	class_weight	['balanced']
	random_state	Seed = 27
Random Forests	n_estimators	Start-stop ranges from 100 to 810 on 10-sized steps (e.g. 100 to 110, 120 to 130, and such).
	criterion	['gini']
	class_weight	['balanced']
	max_features	['auto', 'sqrt']
	max_depth	[10, 20, 30, 40, 50, 60, 70, 80, 90, 100, 110]
	min_samples_split	[2, 5, 10]
	min_samples_leaf	[1, 2, 4]
	bootstrap	[True, False]
	random_state	Seed = 27
	n_jobs	[-1]

Figure S8. Hyperparameter series per classifier, as used in the study.

Classifier	Training Complexity	Prediction Complexity
Linear Classifier with Elastic Net	$O(n \cdot d^2)$	$O(d)$
k-Nearest Neighbour (k-NN)	$O(1)$	$O(n \cdot d)$
Decision Tree	$O(n \cdot d \cdot \log(n))$	$O(\log(n))$
Random Forest	$O(m \cdot n \cdot d \cdot \log(n))$	$O(m \cdot \log(n))$

Figure S9. Here, n is the number of samples and d the number of features. The linear classifier with Elastic Net regularization and the Decision Tree classifier have lower prediction complexity compared to k-NN and Random Forest. The latter incurs higher training and prediction costs due to its ensembling, with m denoting the number of trees. k-NN, although simple and effective, becomes somewhat computationally expensive at prediction time; even if our dataset is not that large, so not so detrimental.

Modality 1

Features dropped		Transformations
place_cook*	healthy_food_lack_minor**	Transform “No”:2 into “No”:0.
kitchen*	low_food_variety_minor**	
food_worry**	food_lack_meal_minor**	cooking_place: place_cook and kitchen results were merged to answer the question “where does the household cook”.
food_ran_out**	ate_less_food_minor**	
healthy_food_lack_adu**	ate_less_meals_minor**	
low_food_variety_adu**	hunger_minor**	
food_lack_meal_adu**	ate_once_minor**	
ate_less_food_adu**	type_fuel***	results_ELCSA: calculation of the food security scale as: food safety, mild insecurity, moderate insecurity, and severe insecurity.
hunger_adu**	type_stove***	
ate_once_adu**		

* Transformed into one feature.
** Used for calculating the ELCSA food security scale and dropped to avoid multi-collinearity.
*** Caused multi-collinearity.

Modality 2

Features dropped	Transformations
exp_hospital*	percentage calculation: the features included were transformed into the percentage of each from the total category expense, e.g.
exp_care*	
exp_alternative_care*	$\% \, of \, exp\_fruits = \frac{exp\_fruits}{total\_food\_expenses}$
exp_dentist*	
exp_medicine*	
exp_prosthesis*	total_food_expense: sum of all features related to food expenses.
exp_tests*	
exp_other_healthcare*	total_health_expense: sum of all features related to health expenses.
exp_insurance*	

* > 50% of the features were reported as zeros, therefore the percentage calculation ended up being NaN values. This could be related to the type of healthcare affiliation of the person.

Modality 3

Features dropped		Transformations
kinship_pediatric*	care_costs**	Transform “No”:2 into “No”:0, and 8 or 9 into NaN.
kinship_random_adult*	speak_spanish**	
age_health_info_adult*	read_write**	
cvd_cerebral_inf_emb**	hc_afill_ISSSTE***	working_hours: compute numerical feature into binary (>40 hrs/week = 1).
present_alc_drinker**	hc_afill_ISSSTE_st***	
treat_cost**	hc_afill_PEMEX***	
hc_pers_explain**	hc_afill_Defensa***	kinship_health_info_adult: categorical computation of the kinship between the random adult and the child or adolescent.
hc_pers_shared_dec**	hc_afill_Marina***	
hc_pers_time**	hc_afill_SSA***	
hc_pers_questions**	hc_afill_IMSS_Pro***	
hc_pers_know_patient**	hc_afill_private***	
time_diagnosis**	hc_afill_other***	age_group_health_info_adult: compute numerical feature into categorical.
care_travel**	hc_afill_none***	
meds_cost**	hc_afill_notknow***	
hc_pers_coordination**		

* Transformed into one feature.
** Contained more than 35% missing values.
*** Caused multi-collinearity.

Modality 4

Features dropped	Transformations
weight1*	Transform 222.2, 88, or 99 values into NaN depending on the feature.
weight2*	
height1*	av_waist: average measurement of waist circumference (waist1/waist2).
height2*	
av_weight*	av_systolic_bp: Average measurement of systolic blood pressure (bp1_sistolic/bp2_sistolic).
av_height*	
waist1*	
waist2*	av_dyastolic_bp: Average measurement of dyastolic blood pressure (bp1_diastolic/bp2_diastolic).
bp1_systolic*	
bp2_systolic*	
bp1_dyastolic*	BMI: calculation of BMI as:
bp2_dyiastolic*	$BMI = \frac{weight}{(height * 0.01)^2}$
hepatitis_B**	
total_chol_value***	
hepatitis_C****	daily_minutes_sitted_down: computation of minutes sitting down in average per day because the original format was “00:00”.
	age_group_health_info_adult: compute numerical feature into categorical.

* Transformed into one feature.
** Contained more than 35% missing values.
*** Caused multi-collinearity.
**** Binary feature.

Modality 5

Features dropped	Transformations
read_dng*	Transform “No”:2 into “No”:0, and 8 or 9 into NaN.
read_nf_table*	
read_ing_list*	Transform questions that evaluated correct answers into binary features responding: was this answer correct?
read_none*	
read_not_know*	
product_A**	kinship_nut_know_adult: categorical computation of the kinship between the adult and the child or adolescent.
product_B**	
product_C**	
compare_choice***	age_group_nut_know_adult: compute numerical feature into categorical.

* Features were dependent on the answer of another feature whose answers were unclear. The question was: “Do you know if the packaged foods and bottled drinks have information about their nutritional content?" and the answers available were: Yes, No, Do not know, or Do not respond. Hence, there was no way of knowing the difference of the three latest answers.
** Contained more than 35% missing values.
*** Caused multi-collinearity.

Figure S10. Feature engineering summary, per modality.

Hyperparameter series per classifier

Classifier	Hyperparameter	Series
Elastic Net	alpha	[0.0001, 0.00025, 0.0005, 0.00075, 0.001, 0.005, 0.0025, 0.0075, 0.01, 0.05, 0.1, 1]
	n_iter_no_change	[20, 50, 100, 150]
	l1_ratio	[0.049, 0.10, 0.15, 0.20, 0.25, 0.30, 0.35, 0.40, 0.45, 0.50, 0.55, 0.60, 0.65, 0.70, 0.75, 0.80, 0.85, 0.89, 0.95, 0.99]
	penalty	[‘elasticnet’]
	class_weight	[‘balanced’]
	loss	[‘log’]
	max_iter	[2500]
	random_state	Seed = 27
k-NN	n_neigh	Start-stop ranges from 1 to 905 on 5-sized steps (1-5, 10-15, etc.)
	leaf_size	[10, 20, 30]
	algorithm	['ball_tree','brute', 'kd_tree']
	weights	['uniform'],
	metric	['manhattan']
	n_jobs	[-1]
Decision Tree	criterion	['gini', 'entropy']
	splitter	['best', 'random']
	max_depth	Start-stop ranges from 5 to 55 on 5-sized steps (e.g. 5 to 10, 15 to 20, and such).
	min_samples_split	[0.1, 0.2, 0.3, 0.4, 0.5, 0.6, 0.7, 0.8, 0.9, 1.0]
	min_samples_leaf	[0.1, 0.2, 0.3, 0.4, 0.5]
	max_features	Number of max features in the given dataset.
	class_weight	['balanced']
	random_state	Seed = 27
Random Forests	n_estimators	Start-stop ranges from 100 to 810 on 10-sized steps (e.g. 100 to 110, 120 to 130, and such).
	criterion	['gini']
	class_weight	['balanced']
	max_features	['auto', 'sqrt']
	max_depth	[10, 20, 30, 40, 50, 60, 70, 80, 90, 100, 110]
	min_samples_split	[2, 5, 10]
	min_samples_leaf	[1, 2, 4]
	bootstrap	[True, False]
	random_state	Seed = 27
	n_jobs	[-1]

Figure S11. Hyperparameter tuning values, per classifier.

Feature engineering summary of Modality 1

**Features dropped**		**Transformations**
place_cook*	healthy_food_lack_minor**	Transform "No":2 into "No":0.
kitchen*	low_food_variety_minor**	
food_worry**	food_lack_meal_minor**	<u>cooking_place:</u> place_cook and kitchen results were merged to answer the question "where does the household cooks".
food_ran_out**	ate_less_food_minor**	
healthy_food_lack_adu**	ate_less_meals_minor**	
low_food_variety_adu**	hunger_minor**	
food_lack_meal_adu**	ate_once_minor**	
ate_less_food_adu**	type_fuel***	<u>results_ELCSA:</u> calculation of the food security scale as: food safety, mild insecurity, moderate insecurity, and severe insecurity.
hunger_adu**	type_stove***	
ate_once_adu**		

* Transformed into one feature.
** Used for calculating the ELCSA food security scale and dropped to avoid multi-collinearity.
*** Caused multi-collinearity.

Feature engineering summary of Modality 2

**Features dropped**	**Transformations**
exp_hospital*	<u>percentage calculation:</u> the features included were transformed into the percentage of each from the total category expense. E.g.
exp_care*	$\% \; of \; exp\_fruits = \frac{exp\_fruits}{total\_food\_expenses}$
exp_alternative_care*	
exp_dentist*	
exp_medicine*	
exp_prosthesis*	<u>total_food_expense:</u> sum of all features related to food expenses.
exp_tests*	
exp_other_healthcare*	<u>total_health_expense:</u> sum of all features related to health expenses.
exp_insurance*	

* > 50% of the features were reported as zeros, therefore the percentage calculation ended up being NaN values. This could be related to the type of healthcare affiliation of the person.

Feature engineering summary of Modality 3

**Features dropped**		**Transformations**
kinship_pediatric*	care_costs**	Transform "No":2 into "No":0, and 8 or 9 into NaN.
kinship_random_adult*	speak_spanish**	
age_health_info_adult*	read_write**	
cvd_cerebral_inf_emb**	hc_afill_ISSSTE***	<u>working_hours:</u> compute numerical feature into binary (>40 hrs/week = 1).
present_alc_drinker**	hc_afill_ISSSTE_st***	
treat_cost**	hc_afill_PEMEX***	
hc_pers_explain**	hc_afill_Defensa***	kinship_health_info_adult: categorical computation of the kinship between the random adult and the child or adolescent.
hc_pers_shared_dec**	hc_afill_Marina***	
hc_pers_time**	hc_afill_SSA***	
hc_pers_questions**	hc_afill_IMSS_Pro***	
hc_pers_know_patient**	hc_afill_private***	
time_diagnosis**	hc_afill_other***	age_group_health_info_adult: compute numerical feature into categorical.
care_travel**	hc_afill_none***	
meds_cost**	hc_afill_notknow***	
hc_pers_coordination**		

* Transformed into one feature.
** Contained more than 35% missing values.
*** Caused multi-collinearity.

Figure S12. Feature engineering summary of Modality 1-3.

Feature engineering summary of Modality 4

Features dropped	Transformations
weight1*	Transform 222.2, 88, or 99 values into NaN depending on the feature.
weight2*	
height1*	av_waist: average measurement of waist circumference (waist1/waist2).
height2*	
av_weight*	av_systolic_bp: Average measurement of systolic blood pressure (bp1_sistolic/bp2_sistolic).
av_height*	
waist1*	
waist2*	av_dyastolic_bp: Average measurement of dyastolic blood pressure (bp1_diastolic/bp2_diastolic).
bp1_systolic*	
bp2_systolic*	
bp1_dyastolic*	BMI: calculation of BMI as: $BMI = \frac{weight}{(height * 0.01)^2}$
bp2_dyiastolic*	
hepatitis_B**	
total_chol_value***	
hepatitis_C****	daily_minutes_sitted_down: computation of minutes sitting down in average per day because the original format was “00:00”.
	age_group_health_info_adult: compute numerical feature into categorical.

* Transformed into one feature.
** Contained more than 35% missing values.
*** Caused multi-collinearity.
**** Binary feature.

Feature engineering summary of Modality 5

Features dropped	Transformations
read_dng*	Transform “No”:2 into “No”:0, and 8 or 9 into NaN.
read_nf_table*	
read_ing_list*	Transform questions that evaluated correct answers into binary features responding: was this answer correct?
read_none*	
read_not_know*	
product_A**	kinship_nut_know_adult: categorical computation of the kinship between the adult and the child or adolescent.
product_B**	
product_C**	
compare_choice***	age_group_nut_know_adult: compute numerical feature into categorical.

* Features were dependant on the answer of another feature whose answers were unclear. The question was: “Do you know if the packaged foods and bottled drinks have information about their nutritional content?" and the answers available were: Yes, No, Do not know, or Do not respond. Hence, there was no way of knowing the difference of the three latest answers.
** Contained more than 35% missing values.
*** Caused multi-collinearity.

Figure S13. Feature engineering summary of Modality 4-5.

Performance metrics for late fusion

Classifier	AUROC (training)	AUROC (validation)
**Maximum Rule**	**0.9904 ± 0.0079**	**0.6340 ± 0.0207**
Sum Rule	0.8122 ± 0.0030	0.5713 ± 0.0117
Product Rule	0.3001 ± 0.0000	0.0604 ± 0.0000
Weight Criterion	0.7831 ± 0.0034	0.5846 ± 0.0124
Rule-based	0.5731 ± 0.0054	0.5618 ± 0.0159

Figure S14. Late fusion meta-classifier results.
